# Anion insertion enhanced electrodeposition of robust metal hydroxide/oxide electrodes for oxygen evolution

**DOI:** 10.1038/s41467-018-04788-3

**Published:** 2018-06-18

**Authors:** Zhenhua Yan, Hongming Sun, Xiang Chen, Huanhuan Liu, Yaran Zhao, Haixia Li, Wei Xie, Fangyi Cheng, Jun Chen

**Affiliations:** 1 Key Laboratory of Advanced Energy Materials Chemistry (Ministry of Education), College of Chemistry, Nankai University, Tianjin, 300071 China; 2 Collaborative Innovation Center of Chemical Science and Engineering, Nankai University, Tianjin, 300071 China

## Abstract

Electrochemical deposition is a facile strategy to prepare functional materials but suffers from limitation in thin films and uncontrollable interface engineering. Here we report a universal electrosynthesis of metal hydroxides/oxides on varied substrates via reduction of oxyacid anions. On graphitic substrates, we find that the insertion of nitrate ion in graphene layers significantly enhances the electrodeposit–support interface, resulting in high mass loading and super hydrophilic/aerophobic properties. For the electrocatalytic oxygen evolution reaction, the nanocrystalline cerium dioxide and amorphous nickel hydroxide co-electrodeposited on graphite exhibits low overpotential (177 mV@10 mA cm^−2^) and sustains long-term durability (over 300 h) at a large current density of 1000 mA cm^−2^. In situ Raman and operando X-ray diffraction unravel that the integration of cerium promotes the formation of electrocatalytically active gamma-phase nickel oxyhydroxide with exposed (003) facets. Therefore, combining anion intercalation with cathodic electrodeposition allows building robust electrodes with high electrochemical performance.

## Abstract (web summary)

Electrodeposition provides a facile fabrication means for electrochemical devices but weak substrate-deposit interactions cause poor performance. Here, authors utilize anion insertion within graphitic layers to improve the material interfaces and construct highly active O_2_-evolving electrocatalysts.

## Introduction

Electrodeposition is a conventional, useful technology to prepare thin films of functional materials such as metal/alloy plating and oxide/chalcogenide semiconductors^1–6^. In particular, there is an increasing interest in direct electrodepositing electrochemically active materials on conducting substrates for electrocatalysis, battery, and supercapacitor applications^7–10^. The electrodeposited freestanding electrodes possess advantageous active site utilization and simple fabrication over the conventional powder form, which is physically mixed with polymeric binder and conducting agent to make a slurry for coating on current collector^11^. However, electrosynthesis is often limited in the formation of thin film due to weak deposit–substrate interaction, insufficient mass transport to the electrode surface, unwanted blocking of evolved gas, low nucleation rate, and high resistance of the deposit^12–15^. Despite progress in fabrication of thick layer on three-dimensional (3D) porous matrix (e.g., Zn, MnO_2_/Ni foam, and Nb_2_O_5_/graphene)^16–18^, it is difficult to achieve high loading on flat substrates and the substrate–deposit interface effect remains elusive. Furthermore, electrodeposition of a target material proceeds via a specific anodic or cathodic reaction involving selected precursor, posing great challenge in universality of the synthesis.

The oxygen evolution reaction (OER) plays a key role in developing clean energy conversion and storage technologies, as it generates electrons that can reduce water to hydrogen, carbon dioxide to carbon-containing fuels, and metal ions to metal in recharge of metal–air batteries^19–22^. Industrial application of OER-related devices relies on the use of low-cost, abundant and efficient electrocatalysts to promote the sluggish process involving four-electron transfer^8,23^. Alternative to the state-of-the-art Ru and Ir-based materials, substantial advances have been made in exploiting nonprecious oxygen-evolving catalysts, among which some first-row transition metal hydr(oxy)oxides/oxides show superior intrinsic activity^24–29^. Ultrathin film electrodes exhibit high gravimetric specific activity but cannot afford large current due to the limited amount of active sites. Increasing mass loading is a feasible way to achieve high current at moderate overpotentials^30^ but would hamper mass transport and charge transfer. Another difficulty to operate at high OER currents concerns the rigorous bubble release, which causes bubble-shielding effect and catalyst peeling off problem^7,31^. There remains formidable challenge in improving the activity and robustness of OER catalysts at current densities up to 1000 mA cm^−2^. It is desirable to build self-supporting electrodes with high catalyst loading, abundant active sites, and super hydrophilic/aerophobic properties by interfacial engineering.

Here, we report a general strategy to incorporate a variety of metal (including IIA, IIB, IIIB, VIB, and VIII elements) hydroxides/oxides into graphitic substrates by leveraging anion intercalation and cathodic electrodeposition. Various metal hydroxides/oxides can be synthesized by increasing cathode surface pH with oxyacid anions reduction. The co-electrodeposition of Ni hydroxide and Ce oxide on graphite support generates robust and homogeneous catalyst layers through anion insertion in graphene layers, eliminating the need of binders. As an exemplified application, NiCeO_*x*_H_*y*_/graphite serves as an electrocatalytic electrode with high OER performance surpassing the state-of-the-art RuO_2_ benchmark and superior stability at large current densities. A combination of in situ Raman and operando X-ray diffraction (XRD) further reveals that electrocatalytically active γ-NiOOH with exposed (003) facets forms on NiCeO_*x*_H_*y*_ at low overpotentials. These findings would enlighten the interface-controllable electrosynthesis of advanced electrodes viable to industrial applications.

## Results

### Anion intercalation enhanced electrodeposition synthesis

To synthesize metal hydroxides, we applied the cathodic electrodeposition, which has been widely used in plating metal coatings. Cathodic reactions such as oxyacid anion reduction generate hydroxide ions (e.g., Eqs. (1)–(3)) and give rise to an increase of pH value close to the cathode surface. For most metals, the standard potentials (*E*^*θ*^) of reactions (1–3) are higher than those of the cation reductions. Based on Pourbaix diagram (example of Ni species shown in Supplementary Fig. 1)^32^, the generated species is thermodynamically controlled by parameters of applied potential, cation concentration, and pH value. Cathodic reduction increases the local pH value near the electrode and thereby kinetically drives the deposition of metal hydroxides and in some cases metal oxides when hydroxide is further dehydrated or oxidized in air. Notably, the cathodic electrosynthesis also favors the coprecipitation of bi-metallic or multi-metallic hydroxides.

1 $${\mathrm{ClO}}_3^ - + 3{\mathrm{H}}_2{\mathrm{O}} + 6{\mathrm{e}}^ - \to {\mathrm{Cl}}^ - + 6{\mathrm{OH}}^ - \quad E^\theta = 1.890\,{\mathrm{V}}$$

2 $${\mathrm{IO}}_3^ - + 3{\mathrm{H}}_2{\mathrm{O}} + 6{\mathrm{e}}^ - \to {\mathrm{I}}^ - + 6{\mathrm{OH}}^ - \quad E^\theta = 1.088\,{\mathrm{V}}$$

3 $${\mathrm{NO}}_3^ - + {\mathrm{H}}_2{\mathrm{O}} + 2{\mathrm{e}}^ - \to {\mathrm{NO}}_2^ - + 2{\mathrm{OH}}^ -\quad E^\theta = 0.838\,{\mathrm{V}}$$

**Fig. 1:**
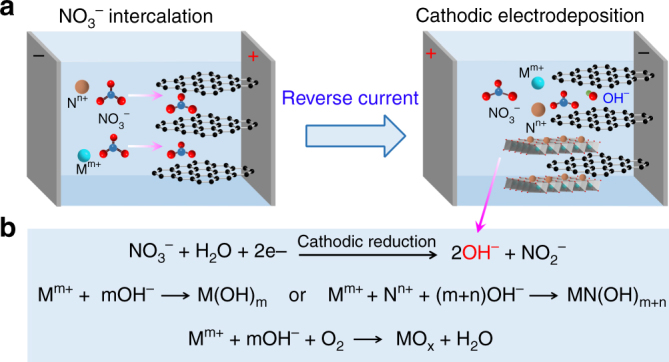
Electrodeposition strategy and principles. **a** A schematic two-step synthesis using graphite (G) substrate and nitrate precursors. **b** The reaction mechanisms of the cathodic electrodeposition of metal hydroxides (M(OH)_m_ or MN(OH)_m+n_), and oxides (MO_*x*_). M^m+^ and N^n+^ are metal cations

The substrate plays a prominent effect on the interface property and the mass loading of the metal hydroxide deposit. Graphitic materials are found to be particularly beneficial in present cathodic electrodeposition as the graphene interlayer accommodates anions^33,34^ and thereby enhances substrate–deposit interaction. To take advantage of the anion intercalation, we adopted a two-step deposition, as illustrated in Fig. 1a. First, a positive potential was applied to facilitate the insertion of NO_3_^−^ ions into the graphite lattice via electrostatic attraction. Then, the current was reversed to allow cathodic reduction and simultaneous electrodeposition (Fig. 1b), which resulted in the generation of graphite-supported metal hydroxide film. Intercalation of NO_3_^−^ in graphite was evidenced by a combination of XRD, Fourier-transform infrared spectroscopy (FTIR), and Raman analysis (Supplementary Fig. 2), which revealed a shift of (002) diffraction peak with intensity decrease, the presence of characteristic NO_3_^−^ signature at 1380 cm^−1^, as well as the peak shift of G band from 1585 to 1605 cm^−1^ and the appearance of D band at 1355 cm^−1^, respectively. The intercalation of metal cation could be negligible in anodic treatment at positive potentials due to electrostatic repulsion but would favor the generation of metal hydroxide in the cathodic electrodeposition.

**Fig. 2:**
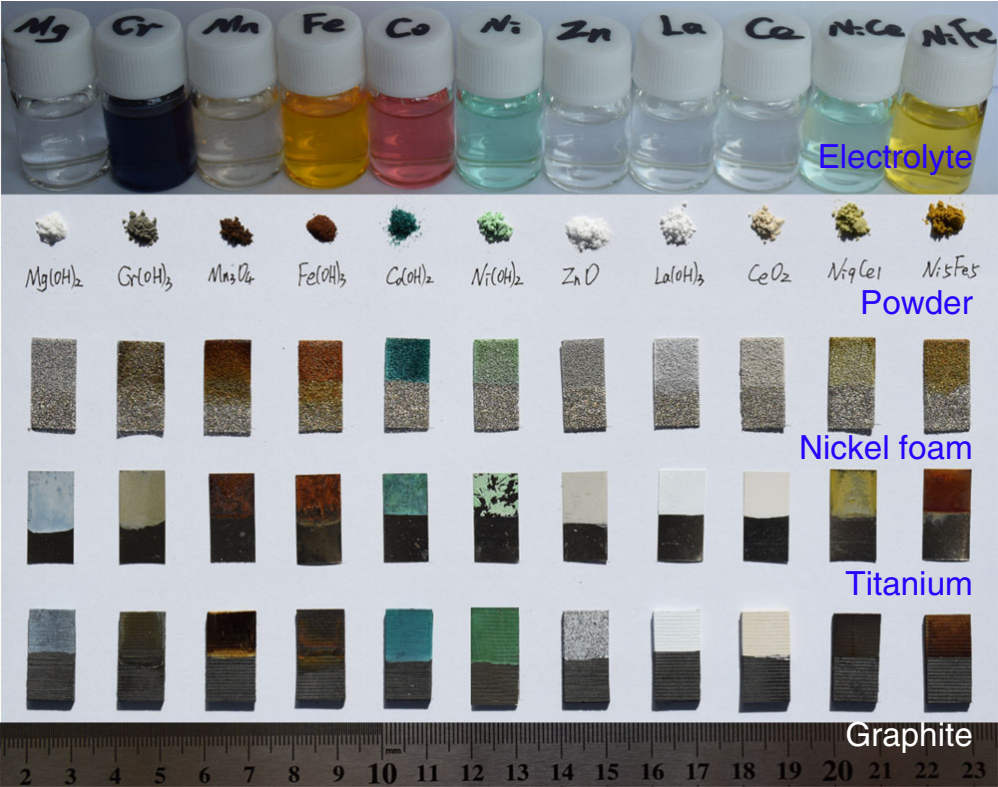
Universality of the cathodic electrodeposition. Optical photographs of the electrolyte precursors, the corresponding generated samples on different substrates, and the collected powders from Ti foil. Each electrodeposition was performed by applying a constant current density of −20 mA cm^−2^ for 300 s in 0.1 M metal nitrate electrolyte

The cathodic electrodeposition is a general, efficient strategy to prepare hydroxides of different metals on a variety of conducting substrates. We selected alkaline earth metal (Mg), 3*d* transition metals (Cr, Mn, Fe, Co, Ni, and Zn), and lanthanides (La and Ce) as examples to demonstrate the universality of the synthesis. Figure 2 shows the digital photos of a series of 0.1 M metal nitrate solutions and the corresponding deposited samples. Powder XRD (Supplementary Fig. 3) analysis indicates the formation of crystalline hydroxides/oxides, including Mg(OH)_2_ (Joint Committee on Powder Diffraction Standards JCPDS #7-239), Cr(OH)_3_ (#12-241), Co(OH)_2_ (#30-443), La(OH)_3_ (#83-2034), Mn_3_O_4_ (#16-154), ZnO (#89-511), and CeO_2_ (#81-792). Other hydroxides such as the obtained Ni(OH)_2_, Fe(OH)_3_, and bimetallic Ni–Ce (denoted as NiCeO_*x*_H_*y*_) feature poor crystallinity or amorphous form. The morphology and size of the electrodeposited samples can be viewed from scanning electron microscopy (SEM) images (Supplementary Fig. 4), which present different shapes including nanosheets (Mg(OH)_2_, Co(OH)_2_, and NiFeO_*x*_H_*y*_) and aggregated nanoparticles (Cr(OH)_3_, Mn_3_O_4_, Fe(OH)_3_, ZnO, and La(OH)_3_).

Benefiting from the cathode reduction process without oxidizing corrosion, common metals (e.g., Fe, Cu, Ni, and Ti) and electroconductive materials (e.g., carbon, indium tin oxide, and fluorine-doped tin oxide) can serve as the substrates. We comparatively demonstrate the electrodeposition of metal hydroxides/oxides on 3D (carbon paper CP and nickel foam NF) and two-dimensional (2D) (Ti and graphite sheet) supports. The deposits show super hydrophilicity, in contrast with the hydrophobic properties of the substrates (Supplementary Fig. 5). On flat Ti substrate, powdery samples can be easily scraped off from the surface. On 3D NF and CP substrates, a pre-ultrasonication treatment in electrolyte results in uniform coating and deep electrodeposition inside the porous skeleton (Supplementary Fig. 6). Interestingly, firm deposit adherence is observed on graphite substrate, which differs from weak binding on other smooth surfaces such as glass carbon (GC), copper, and conducting tin oxides.

### Characterization of electrodeposited hydroxides/oxides

**Fig. 3:**
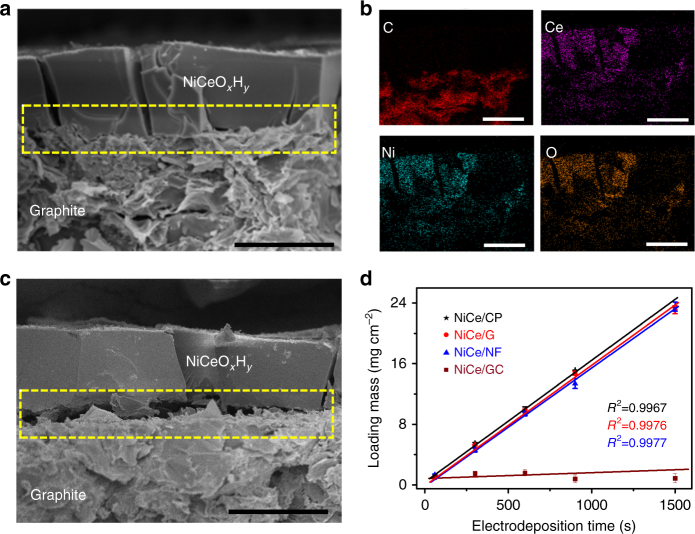
Enhanced electrodeposition on nitrate-inserted graphite. **a** Cross-section SEM image of the prepared NiCeO_*x*_H_*y*_ deposited on graphite after nitrate ion insertion. **b** Elemental mapping of C, Ce, Ni, and O. **c** Cross-section SEM image of NiCeO_*x*_H_*y*_ directly deposited on graphite without foregoing anion intercalation. Scale bars, 25 μm. **d** Relationship between the electrodeposition time and the loading mass of NiCeO_*x*_H_*y*_ deposited on carbon paper (CP), graphite (G), nickel foam (NF), and glass carbon (GC). The electrodeposition was performed at a current density of −20 mA cm^−2^ in electrolyte containing totally 0.1 M Ni and Ce nitrates

In a representative example of Ni–Ce codeposition, the generated deposit was firmly supported on the graphite substrate without apparent interspace, as shown in the SEM image (Fig. 3a). Elemental mapping clearly showed penetration of Ni, Ce, and O into graphite (Fig. 3b). The firm deposit–substrate interaction was further evidenced by high-resolution transmission electron microscope (HRTEM) imaging (Supplementary Fig. 7), which showed anchoring of NiCeO_*x*_H_*y*_ nanoparticles on graphite layers. In comparison, the electrodeposition without a foregoing anion-intercalation process generated a clear gap between the hydroxide deposit and the graphite substrate (Fig. 3c and Supplementary Fig. 8). This shaky deposit film would hinder the electrical conductivity and mechanical robustness. Electrochemical impedance spectroscopy suggested a drastically lower interface resistance of the NiCeO_*x*_H_*y*_/graphite electrode because of the interface enhancement by nitrate insertion in the substrate (Supplementary Fig. 9).

Another virtue of the nitrate-assisted cathodic electrodeposition is the capability to attain high mass loading of deposits, circumventing the thin film limitation in conventional electrochemical synthesis. The amount of deposit could be easily controlled by electrodeposition time at a constant applied current density. As shown in Fig. 3d, for Ni and Ce co-deposition on 2D graphite as well as 3D porous NF and CP substrates, the loading mass increased linearly to 23 mg cm^−2^ with deposition time. For graphite substrate, the loading mass (*m* in mg) and deposition time (*t* in s) follows a linear relationship of *m* = 0.01599*t* + 0.3051. Similar behaviors were observed for the deposition of individual Ni or Ce hydroxide/oxide (Supplementary Fig. 10). High mass loading along with controllable deposition is desirable for scaled-up production and applications in electrochemical devices such as electrolyzers, supercapacitors and batteries. Note that this cathodic electrodeposition on smooth non-graphitic 2D substrates (such as GC, Ti, Cu, and conductive glass) remains limited in mass loading control due to weak deposit–support adhesion in the absence of anion intercalation.

**Fig. 4:**
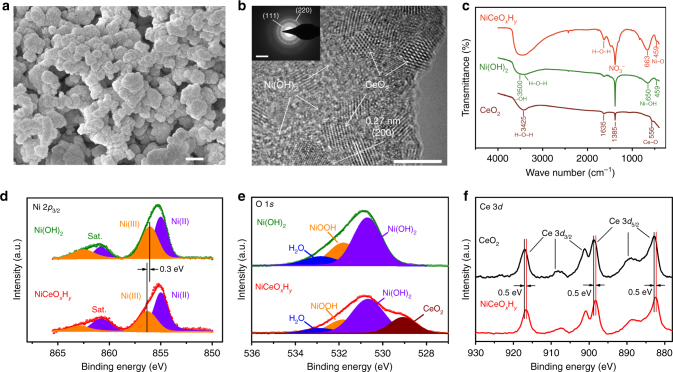
Materials characterization of electrodeposits. **a** SEM (scale bar: 200 nm) and **b** HRTEM (scale bar: 5 nm) images of NiCeO_*x*_H_*y*_. The inset shows SAED pattern (scale bar: 5 1/nm). **c** FTIR spectra of powdery NiCeO_*x*_H_*y*_, Ni(OH)_2_, and CeO_2_. **d**, **e** XPS spectra of powdery Ni(OH)_2_ and NiCeO_*x*_H_*y*_. **f** Ce 3*d* XPS spectra of CeO_2_ and NiCeO_*x*_H_*y*_

Low-magnification SEM images (Fig. 3a and Supplementary Fig. 11) show interconnected cracks in the electrodeposited electrodes. The formation of cracks results from the evaporation of water trapped in the deposits, as seen from the evolution of optical microscope photos during the air-dry process (Supplementary Fig. 12). Magnified imaging (Fig. 4a) reveals that the deposit layer is composed of aggregated nanoparticles with interparticle pores. This rimous texture favors the infiltration of electrolyte inside the electrode, thereby increasing the utilization of active mass. A combination of HRTEM (Fig. 4b and Supplementary Fig. 13), selected-area electron diffraction (SAED) (Fig. 4b, inset) and XRD (Supplementary Fig. 14a) further indicates that the deposited NiCeO_*x*_H_*y*_ hybrid consists of ultrasmall crystalline CeO_2_ nanoparticles (typical size 3.5 nm) and amorphous Ni(OH)_2_. The uniform distribution of nanocrystalline CeO_2_ in Ni(OH)_2_ matrix forms a mosaic structure. The composition of the NiCeO_*x*_H_*y*_ hybrid can be well regulated by adjusting the feeding Ni:Ce ratio in the precursor electrolyte. In a typical hybrid sample, a Ni:Ce atomic ratio of 9:1 was estimated by energy dispersive spectroscopy (EDS) (Supplementary Fig. 14b). Thermogravimetric analysis further indicates that water accounts for 9.9 wt% in the NiCeO_*x*_H_*y*_ hybrid (Supplementary Fig. 15).

The FTIR spectra of the Ni, Ce, and Ni–Ce deposits (Fig. 4c) present bands at 3425 and 1630 cm^−1^, which are assigned to the stretching modes of absorbed water^35^. Characteristic peaks of NO_3_^−^ at 1385 cm^−1^ originated from the nitrate-assisted cathodic electrodeposition. The broad band centered at around 3500 cm^−1^ and the weak peak at 1296 cm^−1^ correspond to the –OH stretching vibration of NiCeO_*x*_H_*y*_ and Ni(OH)_2_. There is a blue shift (ca. 13 cm^−1^) of the Ni–OH bending vibration of NiCeO_*x*_H_*y*_ relative to Ni(OH)_2_, which could be attributed to the change of Ni local environment by CeO_2_ hybridization. Raman spectra (Supplementary Fig. 16) show a broad peak in the range of 420–520 cm^−1^, which could be attributed to Ni–O and Ce–O bands^36^. The appearance of a new vibration at ca. 613 cm^−1^ in NiCeO_*x*_H_*y*_ further evidences the Ni–O–Ce interaction.

X-ray photoelectron spectroscopy (XPS) was performed to probe the oxidation states of Ni and Ce in the electrodeposits. As shown in the Ni 2*p*_3/2_ spectra (Fig. 4d), both Ni–Ce hybrid and Ni hydroxide contain Ni(II) (at 855 eV) and Ni(III) species (856.4 and 856.1 eV). There is a positive shift of 0.3 eV for Ni(III) in the Ni–Ce hybrid. Fitting the O 1*s* spectrum (Fig. 4e) of NiCeO_*x*_H_*y*_ shows deconvoluted peaks at 529, 530.6, 531.8, and 532.8 eV, which are assignable to CeO_2_, Ni(OH)_2_, NiOOH, and H_2_O, respectively^37,38^. For Ce 3*d* spectra of CeO_2_ and NiCeO_*x*_H_*y*_ (Fig. 4f), six peaks located at above 881 eV are characteristic of Ce(IV)^39^. All the Ce 3*d* peaks in hybrid NiCeO_*x*_H_*y*_ negatively shifts by 0.5 eV as compared to that of CeO_2_. The positive shift of Ni binding energy along with the negative shift of Ce position indicate electron transfer from Ni to Ce in the Ni–Ce hybrid, which can be interpreted by the higher electron affinity of Ce(IV). The uniform distribution of CeO_2_ and Ni(OH)_2_ phases and firm interparticle contact in the mosaic structure of the hybrid (Fig. 4b) could benefit the Ni–Ce electronic interaction.

### Electrocatalytic OER on electrodeposited electrodes

**Fig. 5:**
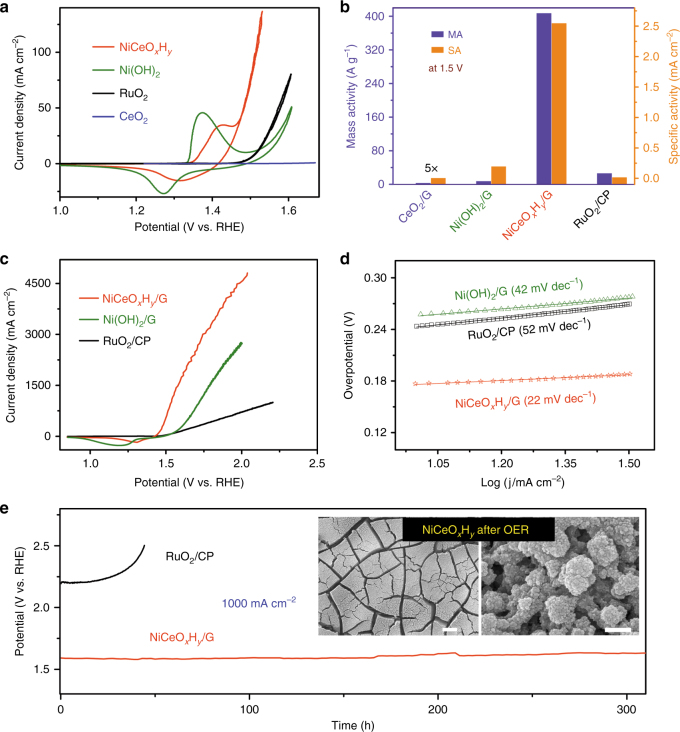
Electrocatalytic OER performance in 1.0 M KOH electrolyte. **a** Voltammetry (scan rate 10 mV s^−1^) of NiCeO_*x*_H_*y*_, Ni(OH)_2_, CeO_2_, and RuO_2_ supported on rotational disk electrode with mass loading of 0.2 mg cm^−2^. **b** Mass and specific activities derived from (**a**) at 1.5 V. The data of CeO_2_/G are multiplied by 5 for clear display. **c** Polarization curves (scan rate 10 mV s^−1^) of NiCeO_*x*_H_*y*_/G, Ni(OH)_2_/G, and RuO_2_/CP electrodes with mass loading of 10, 10, and 6 mg cm^−2^, respectively. **d** Tafel plots derived from the curves in (**c**). **e** Chronopotentiometry of RuO_2_/CP and NiCeO_*x*_H_*y*_/G at a current density of 1000 mA cm^−2^. Inset shows the SEM images (scale bar: left 20 μm, right 200 nm) of the NiCeO_*x*_H_*y*_/G electrode after OER test at 1000 mA cm^−2^ for 300 h

As an exemplified application, the electrodeposited hydroxides were investigated towards the OER electrocatalysis. Figure 5a shows the representative cyclic voltammograms (CVs) of Ni–Ce hybrid, Ni(OH)_2_, CeO_2_, and RuO_2_ supported on graphite-modified GC electrode in rotating disk electrode (RDE) testing mode. The curve of Ni(OH)_2_ presents a redox couple peaked around 1.27/1.37 V and a rapid rising of anodic current at potentials higher than 1.50 V. The redox couple are attributed to the conversion between Ni(OH)_2_ and NiOOH in alkaline electrolytes^40^ while the anodic response corresponds to oxygen evolving. NiCeO_*x*_H_*y*_ displays similar profiles but more overlapping of Ni^2+^ oxidation and OER process. To minimize the masking effect of the preoxidation, we derived the Tafel plots and compared the OER activities using the cathodic sweep.

Remarkably, the NiCeO_*x*_H_*y*_ hybrid exhibited a low onset overpotential of 180 mV and required only 207 mV overpotential to reach a current density of 10 mA cm^−2^ (Fig. 5a). This performance is superior to the benchmark RuO_2_. The difference in OER activity can be further viewed from the Tafel plots of the electrodes (Supplementary Fig. 17). At a given potential of 1.5 V, the mass activity (current density normalized by catalyst mass) of NiCeO_*x*_H_*y*_ is 406.8 A g^−1^, 57 and 15 times that of Ni(OH)_2_ and RuO_2_, respectively (Fig. 5b). The corresponding specific activity (current density based on electrochemically active surface area (ECSA), Supplementary Figs. 18, 19) of NiCeO_*x*_H_*y*_/G is 2.5 mA cm^−2^, 185 times higher than that of the benchmark RuO_2_. While CeO_2_ was almost electrocatalytic inactive, the neat Ni(OH)_2_ electrode showed a considerably low overpotential of 320 mV at 10 mA cm^−2^, which could be attributed to the self-supporting electrode structure that negates the use of binder. Supplementary Fig. 20 clearly reveals the superiority of electrodeposited electrode over the comparative electrodes made by coating a physically mixed powdery catalysts with conducting additive and polymeric binder.

The amount of coated catalyst is limited on GC substrate due to weak affinity. Benefiting from the strong deposit–substrate interaction, the NiCeO_*x*_H_*y*_/G electrode could afford a high mass loading over 15 mg cm^−2^ (Supplementary Fig. 21). At an optimized loading of 10 mg cm^−2^, the electrode exhibits excellent performance (Fig. 5c). A current density of 10 mA cm^−2^ was attained at a low overpotential of 177 mV. A moderate overpotential of 500 mV rendered a large current of 3000 mA cm^−2^. As shown in Fig. 5d, the NiCeO_*x*_H_*y*_/G electrode gave a Tafel slope of 22 mV dec^−1^, much lower than those of Ni(OH)_2_ (42 mV dec^−1^) and RuO_2_ (52 mV dec^−1^). These values were smaller as compared to that measured on RDE, suggesting faster OER kinetics of the electrodeposit on graphite.

Supplementary Table 1 summarizes the detailed performances of some reported state-of-the-art OER catalysts including Au foam-supported FeCoW, GO-supported NiFe-layered double hydroxide (LDH), RuO_2_, IrO_2_, and Ir/C^7,27,41–43^. The comparison indicates that the NiCeO_*x*_H_*y*_/G hybrid outperforms most of the previous advanced OER catalysts, in terms of widely used figures of merit such as onset potential, overpotential at 10 mA cm^−2^, ECSA-based specific activity at overpotential of 270 mV, mass activity, turnover frequency (TOF), and Tafel slope^42^. In a recent report, NiCeO_*x*_ supported on Au exhibited striking activity due to the beneficial effect of Au^29^. We show here Au is not indispensable but instead, graphitic substrates give rise to even better performance in affording Ni–Ce electrodeposit. Additionally, we investigated the effect of Ni:Ce molar ratio on the electrocatalytic performance of the hybrid and found a optimized composition of 9Ni:Ce (Supplementary Fig. 22). Furthermore, electrochemical tests of the electrodeposited NiFeO_*x*_H_*y*_/G and Co(OH)_2_/G samples indicate respectable OER performance of the NiFe hybrid and limited catalytic activity of Co-based hydroxide catalysts (Supplementary Fig. 23).

Besides activity, the long-term durability of OER catalysts at high currents (ca. 1000 mA cm^−2^ level) is required for practical application such as electrolyzers. The NiCeO_*x*_H_*y*_/G with a loading mass of 10 mg cm^−2^ exhibits excellent stability, withstanding constant current of 1000 mA cm^−2^ over 300 h (Fig. 5e). We observed homogenous bubble formation on electrode surface and rigorous gas release without catalyst peeling off (Supplementary Movie 1). The chapped morphology, porous texture, and particle size were well retained after durability testing, inspite of the loss of the mossy particles on the surface of the electrode (Fig. 5e inset and Supplementary Fig. 24). A post compositional investigation of the electrode revealed almost unchanged Ni:Ce ratio in NiCeO_*x*_H_*y*_ (Supplementary Fig. 24d) and undetectable metal leaching in KOH electrolyte after the OER tests. Furthermore, the mechanical stability of NiCeO_*x*_H_*y*_/G electrode was proved by its robustness against ultrasonic treatment (Supplementary Fig. 25).

**Fig. 6:**
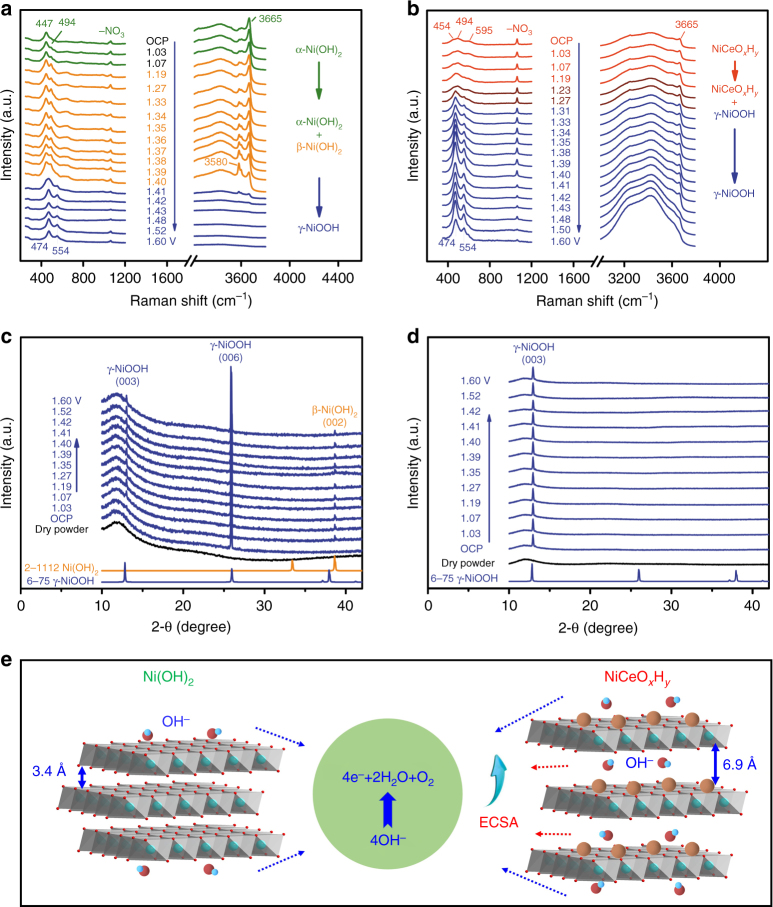
OER mechanism studies. **a**, **b** In situ Raman spectra collected on (**a**) Ni(OH)_2_/G and (**b**) NiCeO_*x*_H_*y*_/G electrodes from open circuit potential (OCP) to 1.6 V (vs. RHE) in 1 M KOH. **c**, **d** Operando XRD patterns collected on (**c**) Ni(OH)_2_/NF and (**d**) NiCeO_*x*_H_*y*_/NF electrodes at different potentials. Data of pristine dry powders and standard profiles of Ni(OH)_2_ and NiOOH are shown for reference. **e** Cartoon showing that the OER proceeds more favorably on NiCeO_*x*_H_*y*_ with larger interlamellar spacing and higher ECSA relative to Ni(OH)_2_

To gain insight into the electrocatalytic behaviors, we performed in situ Raman and XRD analysis of the electrodeposited electrodes in 1 M KOH. Raman spectra reveal a clear change of the electrodes after being immersed in alkaline electrolyte (Fig. 6a, b) as compared to their pristine states (Supplementary Fig. 16). There is a decrease of intensity of NO_3_^−^ peak, suggesting the replacement of NO_3_^−^ with OH^−^. For Ni(OH)_2_ (Fig. 6a), the Ni–O vibration shifts from 463 to 447 cm^−1^ at open circuit potential (OCP). With increasing electrode potential, a new peak emerges at 494 cm^−1^ and its intensity increases, indicative of enhanced crystallinity. At potentials above 1.36 V, the Ni–O vibration weakens while the O–H vibration ascribed to α-Ni(OH)_2_ varies at 3580–3668 cm^−1^. The appearance of the characteristic peak at 3580 cm^−1^ indicates the conversion of α- to β-Ni(OH)_2_^44^. At 1.41 V, Ni(OH)_2_ is transformed into γ-NiOOH, as viewed from the characteristic peaks (474 and 554 cm^−1^)^45^.

Interestingly, NiCeO_*x*_H_*y*_ shows much different spectroscopic and electrochemical behaviors (Fig. 6b). Signals associated with α-Ni(OH)_2_ are almost not discernable. Additionally, the γ-NiOOH phase appears at 1.27 V, 0.14 V lower than the conversion potential of Ni(OH)_2_. These results indicate that γ-NiOOH is the active phase for the OER, in agreement with previous investigations on nickel hydroxides^7,29^. The formation of NiOOH after OER was also proved by the FTIR (Supplementary Fig. 26). While neat CeO_2_ remains unchanged during the OER (Supplementary Fig. 27), the incorporation of Ce in Ni–Ce would reduce the barrier energy of Ni(II)–Ni(III) transformation and result in lower OER overpotentials. The higher Faraday efficiency of OER on NiCeO_*x*_H_*y*_ electrode relative to Ni(OH)_2_ electrode (Supplementary Fig. 28) further suggest the beneficial effect of Ce in promoting the formation of electrocatalytically active Ni(III) species.

To further probe the structural evolution during the OER process, we performed an operando XRD analysis of Ni(OH)_2_ and NiCeO_*x*_H_*y*_. Nickel foam was employed as the catalyst support to exclude the masking effect of the graphitic substrate. In contrast to the amorphous state of pristine Ni(OH)_2_ and NiCeO_*x*_H_*y*_ powders, the electrodes show considerable crystallinity after shortly aging in 1 M KOH electrolyte. At applied potentials, the Ni(OH)_2_ electrode occurs mainly in γ-NiOOH phase with a small portion of β-Ni(OH)_2_ (Fig. 6c). For the NiCeO_*x*_H_*y*_ electrode, only γ-NiOOH phase is discernable (Fig. 6d), in consistent with the Raman analysis. Note that γ-NiOOH exposes merely (003) facets in NiCeO_*x*_H_*y*_, as compared to the preferential (006) planes in Ni(OH)_2_. The interplanar spacings of (003) and (006) planes are 0.69 and 0.34 nm, respectively. We propose that larger lattices favor the ionic intercalation of OH^−^ and the exposure of more active sites for oxygen evolving (Fig. 6e), which is confirmed by the fivefold increase of ECSA from Ni(OH)_2_ to NiCeO_*x*_H_*y*_ (Supplementary Fig. 18f). Besides, for NiCeO_*x*_H_*y*_, the Ni–Ce electronic interaction evidenced from FTIR, Raman, and XPS (Fig. 4c–f and Supplementary Fig. 16) is an important factor in enhancing the catalytic activity of nickel hydroxide, since previous density functional theory calculations have demonstrated that the introduction of Ce can modify the binding energy of intermediates (e.g., HO*, O*, and HOO*) involved in the OER and thereby reduce the overpotentials^28^.

## Discussion

We have developed a universally applicable, environmentally benign, and easily scalable electrosynthesis method to prepare a variety of metal (e.g., alkaline earth metals, 3*d* transition metals and lanthanides) hydroxides/oxides on different substrates. Metal hydroxides are cathodically electrodeposited by increasing local pH via oxyacid anion reduction reactions. In a peculiar nitrate-assisted electrodeposition, graphitic substrates are found to allow support–deposit interface engineering because of anion intercalation into the graphene layers. The synthesis generates hydroxides or bimetallic hydroxide/oxide hybrids on graphite electrodes with strong adherence, rimous texture, superhydrophilicity, and high mass loading up to 23 mg cm^−2^. Particularly, the Ni–Ce hybrid co-deposited on graphite features a mosaic structure that consists of uniformly distributed ultrasmall CeO_2_ nanoparticles in amorphous Ni(OH)_2_ matrix. This strategy may be extended to preparing other functional materials on conducting layer-structured electrodes to reinforce the integration, loading, conductivity, and mechanical robustness, which are prerequisite in electrochemical devices such as electrolyzers, batteries, and supercapacitors. Preliminary investigations on the oxygen reduction reaction properties of Mn_3_O_4_ (Supplementary Fig. 29) and supercapacitive performance of Ni(OH)_2_ (Supplementary Fig. 30) suggest wide applicability of the electrodeposited materials.

As a typical application, we showcase the obtained graphite-supported Ni–Ce composite electrode in catalyzing the technologically important OER reaction. The catalytic performance is superior to the state-of-the-art RuO_2_, IrO_2_, and other reported champion catalysts, exhibiting an overpotential of 177 mV at 10 mA cm^−2^ and sustaining over 300 h stability at large current of 1000 mA cm^−2^. The high activity and durability render the graphite-supported Ni–Ce hybrid a promising inexpensive, yet efficient catalyst for industrial electrolyzer. We further unravel that the integration of Ce in Ni hydroxide modulates the electronic structure of Ni and promotes the formation of γ-NiOOH with preferentially exposed (003) facets that favor the OER due to enhanced ECSA. These findings may aid in the rational design of advanced transition metal hydro(oxy)oxide/oxide electrodes.

## Methods

### Materials

All reagents and solvents used were of analytical grade. Substrates were described as follows: graphite sheet (thickness: 1.0 mm, bulk density: 1.85 g cm^−3^, supplied from Gaofeng), nickel foam (thickness: 1.5 mm, areal density: 0.035 g cm^−2^, Jiayisheng), carbon paper (thickness: 0.20 mm, bulk density: 0.78 g cm^−3^, Toray), titanium sheet (thickness: 0.20 mm, Shuanghua), copper foil (thickness: 0.05 mm, Feintool), indium tin oxide and fluorine-doped tin oxide (thickness: 1.1 mm, Weslsy), and GC sheet (thickness: 1 mm, Aida).

### Materials synthesis

Electrodeposition was performed at 25 °C in a 50 ml electrolytic bath. A conducting substrate (GC, CP, NF, Ti, graphite, etc.), a graphite plate, and a saturated calomel electrode (SCE) served as the working, counter and reference electrode, respectively. The electrolyte was an aqueous nitrate solution containing 0.1 M total metal ions. The bath, graphite plate, and all substrates were ultrasonically cleaned in 1 M HCl and Millipore water (18.2 MΩ cm^−1^). In a typical synthesis, the graphite substrate was first subjected to anodic treatment at 20 mA cm^−2^ for 600 s to allow NO_3_^−^ intercalation and then applied to a cathodic deposition at −20 mA cm^−2^ for 600 s. On non-graphitic substrates such as bare GC, CP, NF, and Ti, direct cathodic electrodeposition was performed at current of −20 mA cm^−2^ for 300 s. For 3D substrates like CP and NF, an ultrasonic pretreatment of 600 s was applied to ensure electrolyte soaking.

For comparison, NiCeO_*x*_H_*y*_ in thin film form was also electrodeposited onto GC electrode following similar procedures described above (current 1.2 mA, duration 55 s). The GC substrate was modified with a graphite layer to enhance adhering of deposit. To prepare graphite-modified GC electrode, 10 mg of graphite powder (Sigma-Aldrich) was dispersed in 950 μl of isopropanol and 50 μl of Nafion solution (5 wt% in water, Sigma-Aldrich). The resultant suspension was then sonicated for 30 min to form a homogenous ink. An aliquot of 5 μl of this ink was afforded on GC and dried in air. The loading amount of deposited NiCeO_*x*_H_*y*_ on GC electrode was around 0.2 mg cm^−2^, as determined by electrochemical quartz crystal microbalance (EQCM).

### Materials characterizations

SEM images were collected with a JEOL JEM-7500F field-emission electron microscope. TEM equipped with EDS and SAED was carried out in a JEOL 2010F 200 keV field-emission electron microscope. Powder XRD measurements were conducted on a Rigaku rotating anode diffractometer with a monochromated Cu Kα X-ray source. Fourier-transform infrared spectra were measured using a Bruker Vertex 70 FT-IR spectrometer. Raman spectra for powder samples were collected using a Horiba LabRAM HR Evolution  microscope with an acquisition time of 20 s. XPS data were collected using a Kratos Axis Ultra DLD spectrometer employing a monochromated Al-Kα X-ray source.

### Electrochemistry tests

Electrochemical measurements were performed on Parstat 4000 potentiostat/galvanostat workstation (AMETEK) and Ivium-n-State multichannel electrochemical analyser (IVIUM), using a three-electrode system with deposited hydroxides, Pt sheet, and Hg/HgO as the working, counter, and reference electrode, respectively. All potentials are reported relative to the reversible hydrogen electrode (RHE) scale unless noted. A consistent solution resistance of 0.5 Ω was measured via electrochemical impedance spectroscopy under OCP and was used for iR correction.

The Faradaic efficiency was calculated from the total amount of oxygen produced and the total charge (*Q*) passed through the electrolytic cell. The total amount of produced oxygen was measured by a drainage method. Assuming that four electrons are needed to produce one O_2_ molecule, the Faradaic efficiency can be calculated as follows^26^:

4 $$\eta _{{\mathrm{O}}_2}{\mathrm{ = }}\frac{{4F \ast {\mathrm{n}}}}{Q}$$

where *F* is the Faraday constant (96,485 C mol^−1^) and *n* is the number of moles of generated O_2_.

EQCM measurements were performed on a QCM922 (Princeton Applied Research) with a two-electrode configuration. An AT-cut platinum-coated quartz crystal of 8.985 MHz resonance frequency with the geometrical area of 0.196 cm^2^ was used as the working electrode, and a platinum plate as the counter electrode. An aqueous solution containing 0.1 M metal ions was used as the electrolyte. The electrodeposition was performed at room temperature in galvanostatic mode at −1.2 mA for 55 s and the corresponding change in resonance frequency was measured. The change in mass per unit area was calculated from the change in resonance frequency using the Sauerbrey equation.

The TOF value was calculated using the following equation^7^:

5 $${\rm{TOF}}{\mathrm{ = }}\frac{{J \ast A}}{{4 \ast F \ast {{m}}}}$$

where *J* (A cm^−2^) is the current density at a given overpotential (e.g., *η* = 270 mV), *A* is the surface area of the electrode (0.2475 cm^2^), *F* is the Faraday constant (96,485 C mol^−1^), and *m* is the number of moles of metal on the electrode. The metal content of Ni(OH)_2_ (56 wt% Ni) and NiCeO_*x*_H_*y*_ (46 wt% Ni and 11.8 wt% Ce) was quantified by ICP-MS. Because Ni is much more active than Ce for the OER catalysis in alkaline solution, Ni atoms are supposed to be the active sites for both Ni(OH)_2_ and NiCeO_*x*_H_*y*_ during the TOF calculation.

### In situ Raman spectra and operando XRD measurements

In situ Raman spectra were acquired under controlled potentials using a homemade cell, which consisted of a working electrode (electrodeposit on 3 mm graphite) at the top, a Pt wire counter electrode and a Ag/AgCl (saturated KCl) reference electrode. Raman spectra were collected using a confocal microscope (Horiba LabRAM HR Evolution) with an excitation wavelength of 633 nm, a power of 1–3 mW, and a spectral resolution of ∼1 cm^−1^. The spot size of the laser beam was estimated to be 1–2 μm. Acquisition time was typically 20 s for the spectral window of 1100 cm^−1^. Spectral shifts were calibrated routinely against the value of a silicon wafer (520.7 cm^−1^). The working electrode was anodically scanned from open circuit potential to 1.6 V at a rate of 0.2 mV s^−1^. Operando XRD measurements were conducted on Rigaku diffractometer at room temperature using the same cell. Diffraction data were collected with speed of 20° min^−1^ at 2*θ* range of 10–43°.

### Data availability

The authors declare that all the relevant data are available within the paper and its Supplementary Information file or from the corresponding author upon reasonable request.

## Electronic supplementary material

Supplementary Information

Description of Additional Supplementary Files 

Supplementary Movie 1
