# Supplementary material for: Anion insertion enhanced electrodeposition of robust metal hydroxide/oxide electrodes for oxygen evolution
Source: Nat Commun. 2018 Jun 18;9:2373. doi: 10.1038/s41467-018-04788-3 (PMC6006371; doi:10.1038/s41467-018-04788-3)
Supplement: Supplementary file 1 — Supplementary Information [file 41467_2018_4788_MOESM1_ESM.pdf]

Supplementary Information

Anion insertion enhanced electrodeposition of robust metal hydroxide/oxide electrodes for oxygen evolution

Yan et al

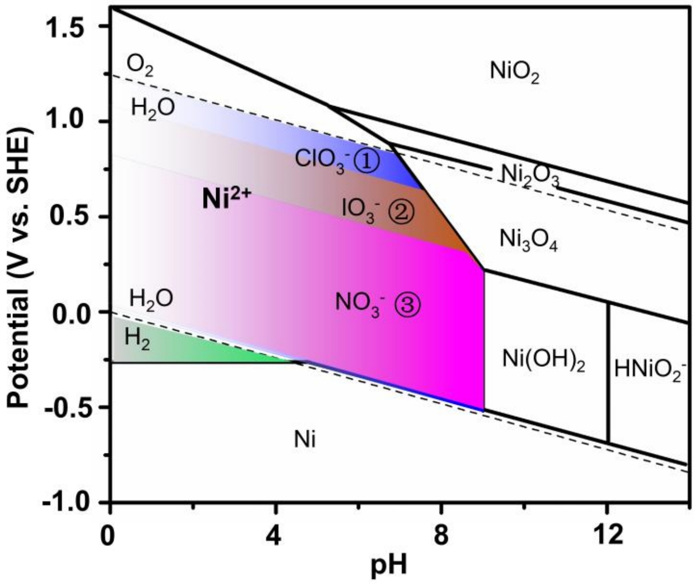

Supplementary Figure 1 | Pourbaix diagram of Ni-based species. The colored regions correspond to the oxoanion reactions that can be applied for the synthesis of nickel hydroxides. The Pourbaix diagram correlates the pH and potential with thermodynamically stable species of an element, which is useful to guide controllable electrosynthesis by adjusting the applied potential and pH of the electrolyte. We present the Pourbaix diagram of nickel species as an example. When using the chlorate as the electrolyte, the hydroxyl ions generated by the reduction of chlorate increase the pH of the cathode surface and allow the formation of metal hydroxides. The nickel hydroxide forms at the potential range of 0–1.23 V, in which we can effectively avoid the oxygen evolution and the reduction of Ni^{2+} to Ni. Similarly, nickel hydroxides will be produced at the applied potential ranges of 0–1.088 and 0–0.838 V when employing the iodate and nitrate electrolytes, respectively.

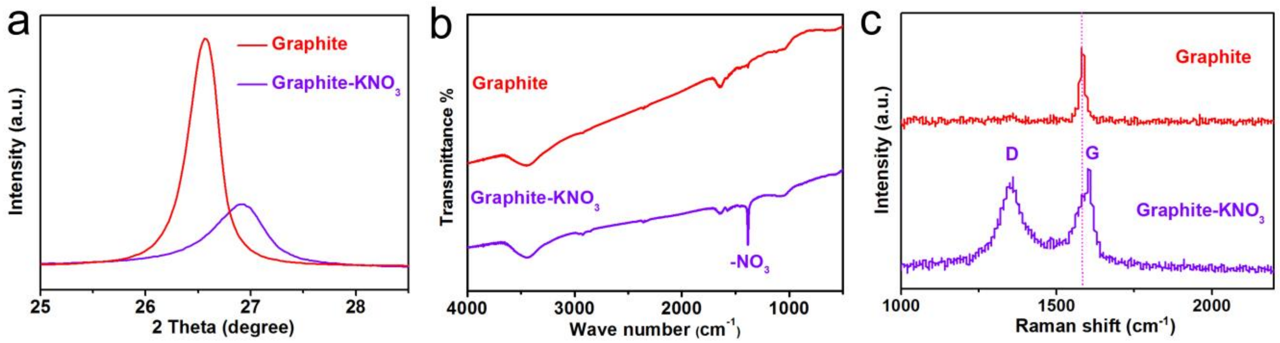

Supplementary Figure 2 | Analysis of nitrate inserted in the graphite substrate. a, XRD **b**, FTIR and **c**, Raman of the pristine and NO_3^- embedded graphite substrates. The graphite was first subjected to anodic oxidation in 0.1 M KNO_3 for 300 s at 20 mA cm^{-2} and then rinsed thoroughly with water to remove the surface-adsorbed nitrate. In the XRD pattern of nitrate-inserted graphite, the intensity of (002) peak decreases significantly as compared to that of the pristine graphite and shifts to a slightly higher 2θ position, indicating a lowering of graphitization degree and a decrease of interlayer spacing after nitrate intercalation. In addition, the FTIR spectrum of treated graphite presents a characteristic peak at 1380 cm^{-1} assignable to the asymmetric stretching vibration of nitrate functional groups[1]. In the Raman spectra of nitrate-inserted graphite, the G band peak shifts from 1585 to 1605 cm^{-1} and an apparent D band peak emerges, which suggest the increase of bonding between graphitic interlayers and the presence of defects and/or structural disorders[2,3].

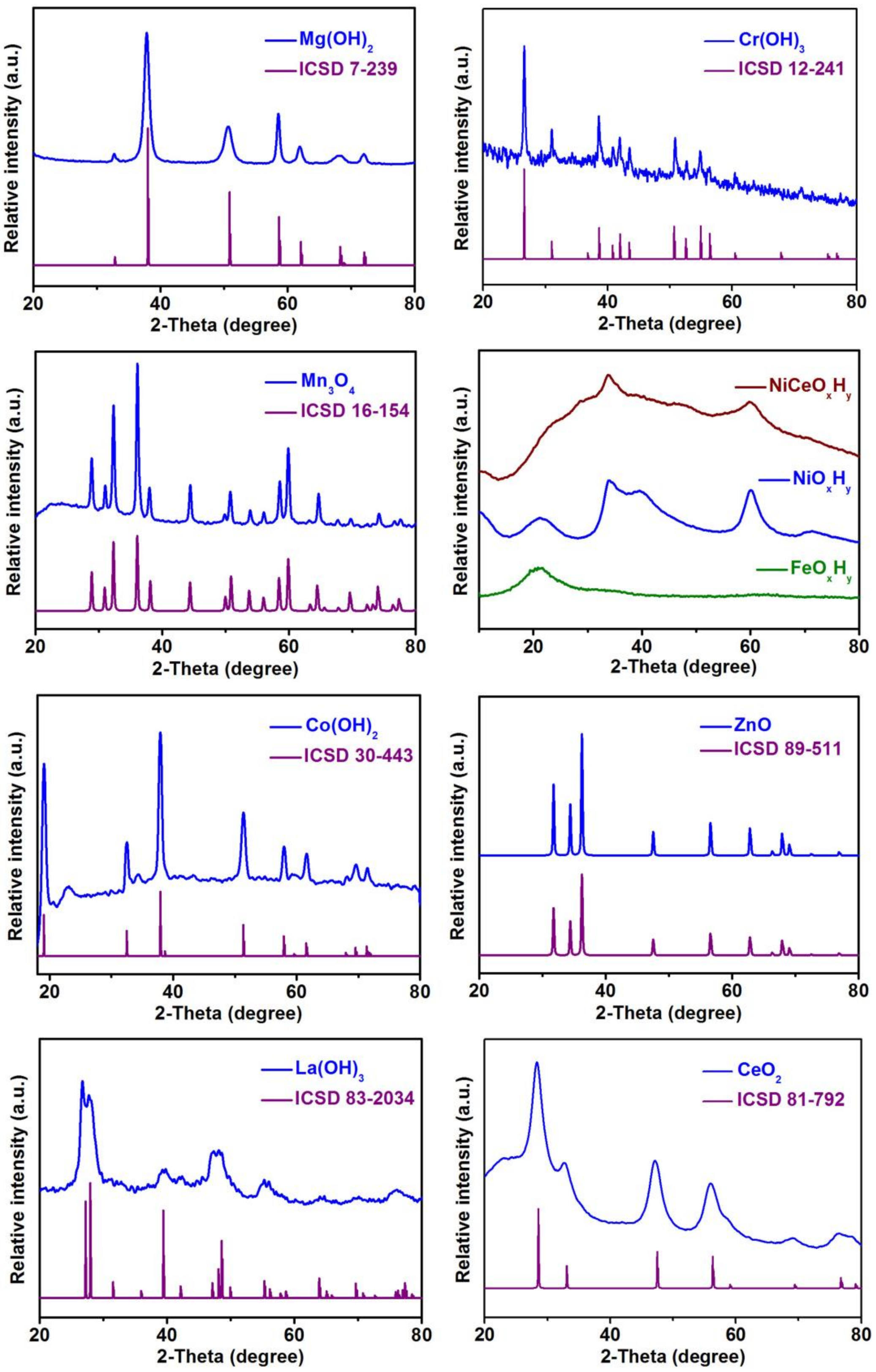

Supplementary Figure 3 | Phase characterization of the deposits. XRD patterns of the powder samples obtained by electrodeposition on Cu or Ti foils.

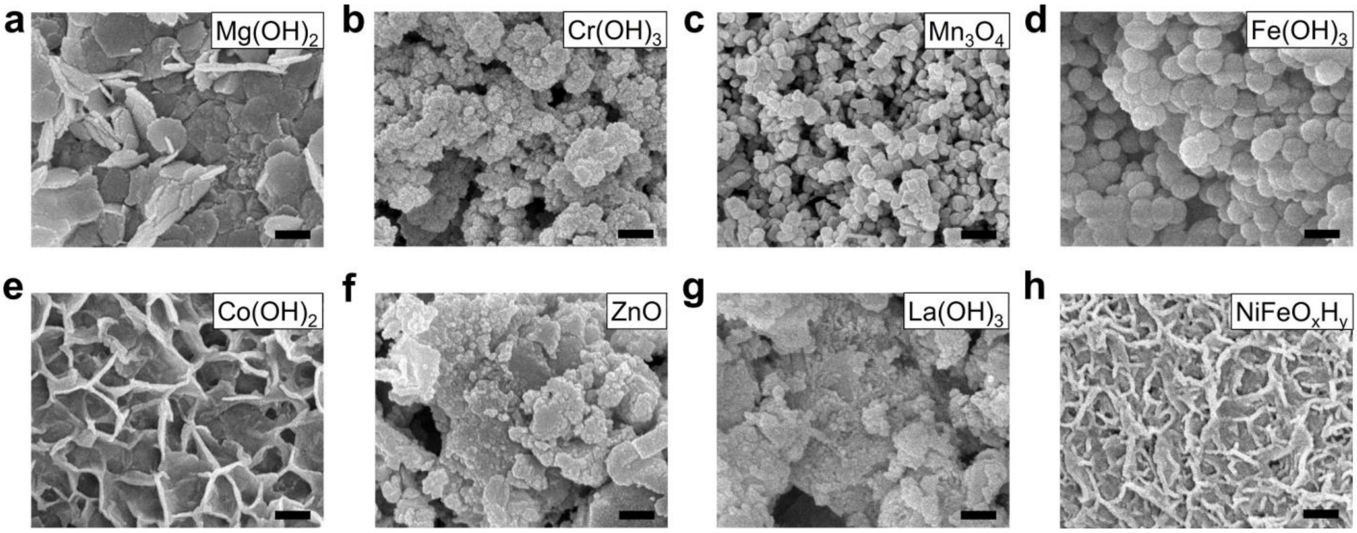

Supplementary Figure 4 | SEM images of the synthesized hydroxides/oxides. **a**, $Mg(OH)_2$. **b**, $Cr(OH)_3$. **c**, Mn_3O_4. **d**, $Fe(OH)_3$. **e**, $Co(OH)_2$. **f**, ZnO. **g**, $La(OH)_3$. **h**, $NiFeO_xH_y$. Scale bars: 200 nm.

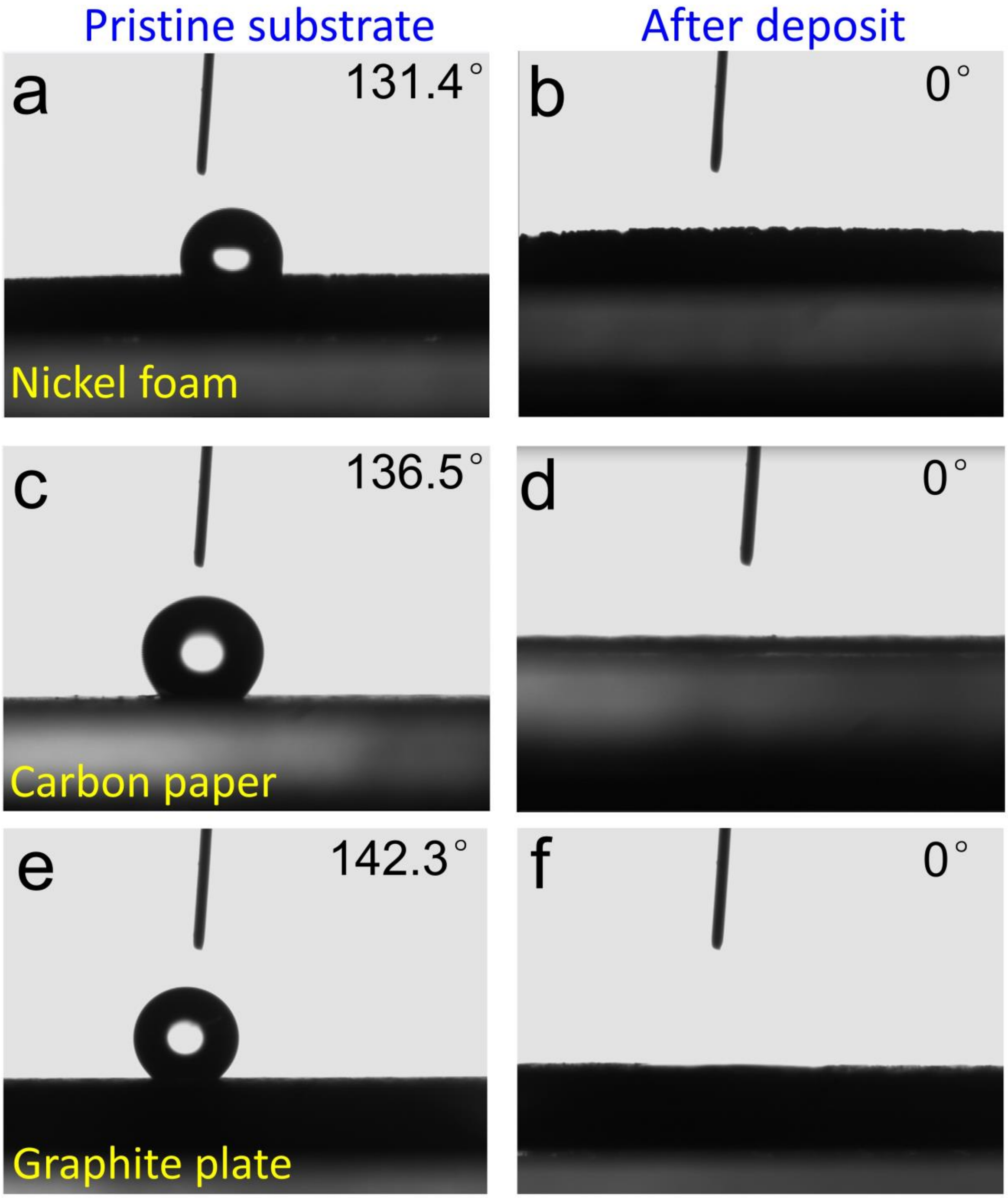

Supplementary Figure 5 | The superhydrophilicity of the deposits. a,**c**,**e**, Contact angles measured on the original substrates. **b**,**d**,**f**, Contact angles on the substrates with electrodeposits of $NiCeO_xH_y$. Photos were recorded after a water droplet on each surface for 5 s.

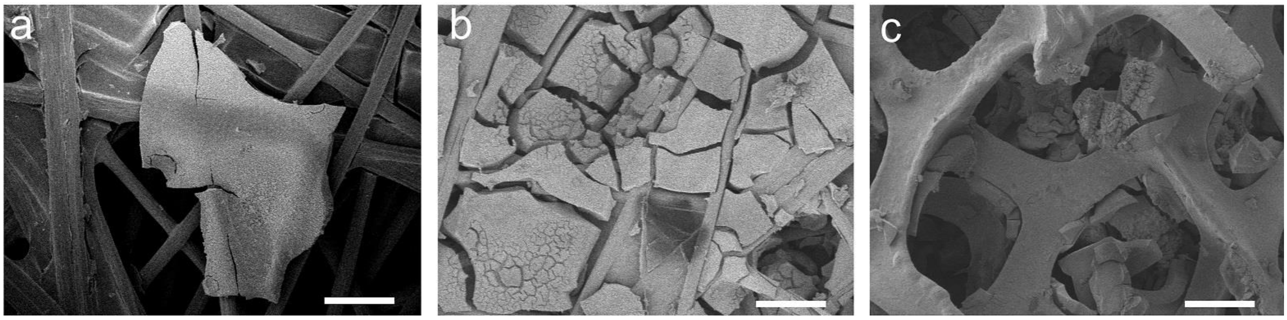

Supplementary Figure 6 | Effect of ultrasonic treatment for the electrodeposition on 3D substrates. SEM images of the $NiCeO_xH_y$. **a**, Directly deposited on CP. Deposited after the ultrasonic treatment on **b**, CP and **c**, NF. Scale bars: **a**,**b**, 40 μm, **c**, 100 μm.

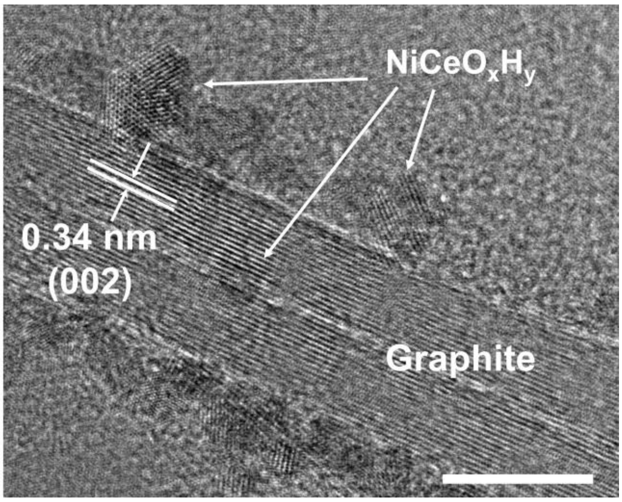

Supplementary Figure 7 | The firm deposit-substrate interaction. HRTEM image of the $NiCeO_xH_y$ deposit in graphite after the insertion of NO_3^- ions. Scale bar: 10 nm.

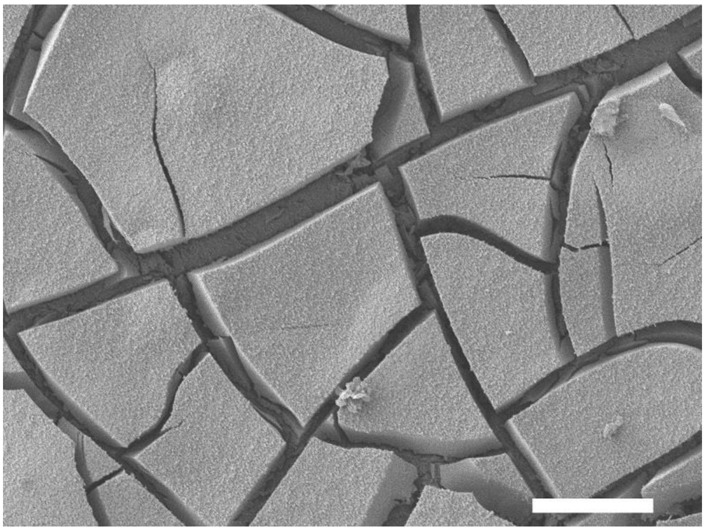

Supplementary Figure 8 | SEM images of $NiCeO_xH_y$/graphite. Top view SEM images of the $NiCeO_xH_y$ deposit on graphite synthesized without a foregoing anodic process to facilitate anion intercalation. Scale bar: 40 μm.

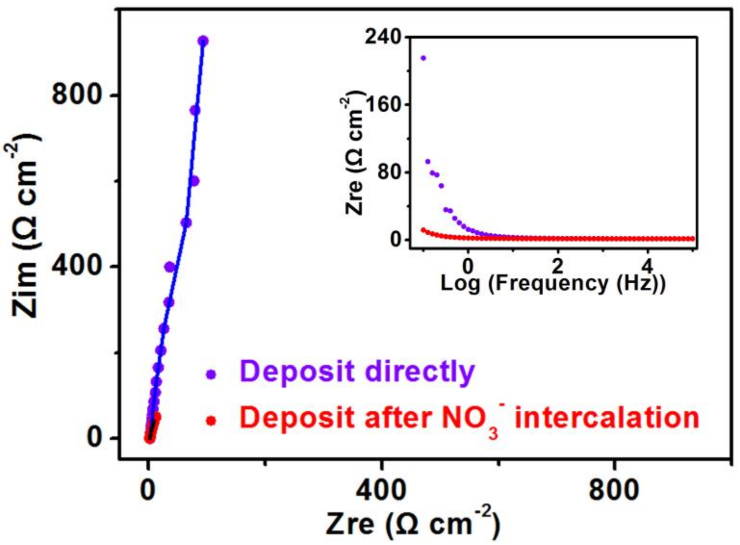

Supplementary Figure 9 | Electrochemical impedance spectroscopies of the Ni-Ce electrodeposits on graphite prepared with and without a foregoing anodic step for nitrate intercalation. Nyquist plots collected at open circuit potential in 1 M KOH with amplitude of 10 mV. Inset shows the Bode plots.

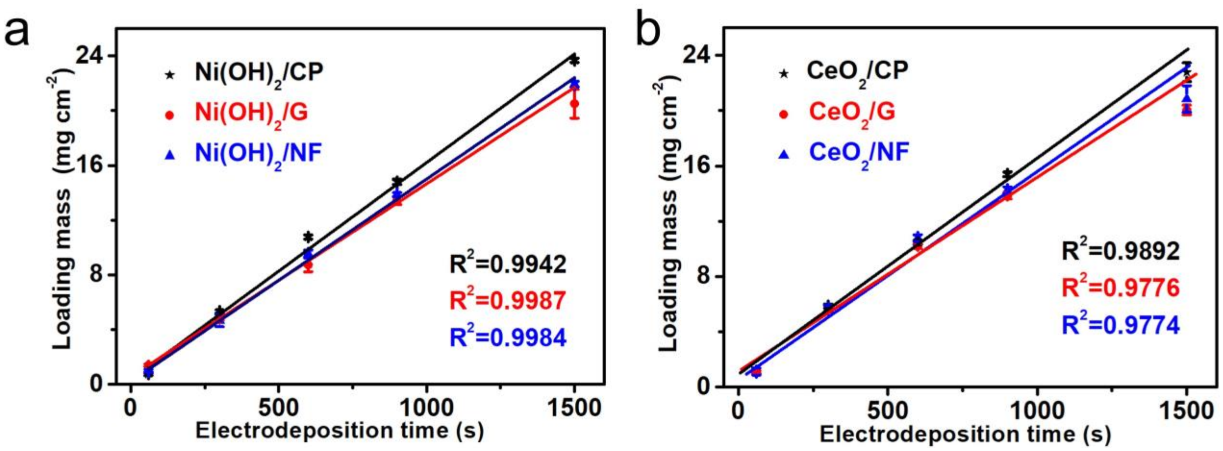

Supplementary Figure 10 | The electrodeposition behaviors of Ni and Ce on different substrates.

The relationship between deposition time and loading mass at various substrates in a solution containing 0.1 M metal nitrate. **a**, Ni and **b**, Ce.

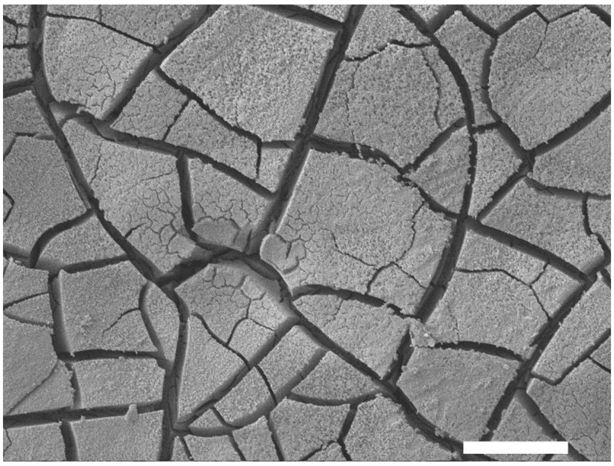

Supplementary Figure 11 | Characterization of the electrodeposits. Top view SEM image of the $NiCeO_xH_y$/graphite samples. Scale bar: 40 μm.

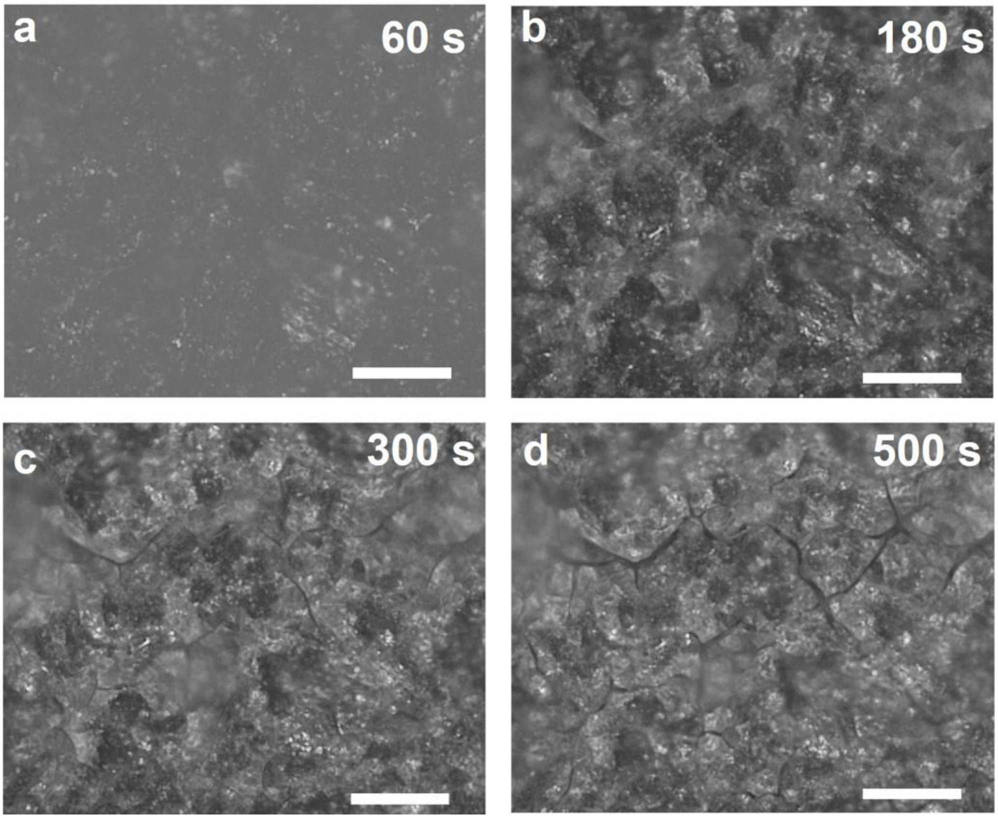

Supplementary Figure 12 | Crack generation in the electrodeposits. Optical microscope photos of the deposit during the air-drying process of **a**, 60 s, **b**, 180 s, **c**, 300 s and **d**, 500 s. In the cathodic electrodeposition, water was trapped in the deposit layers and the interconnected cracks emerged upon water evaporation. Scale bar: 100 μm.

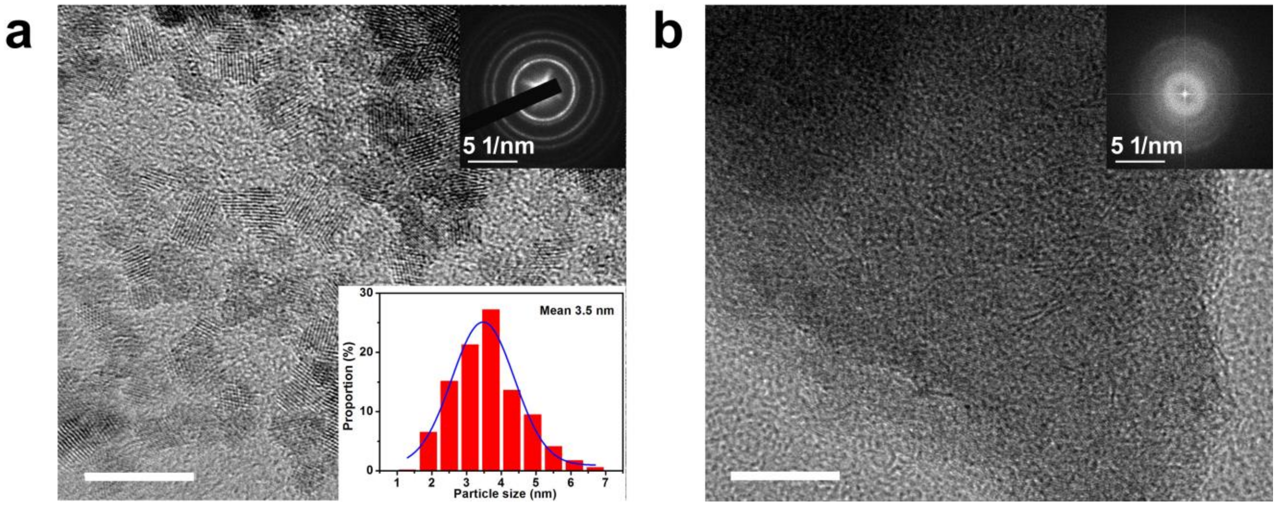

Supplementary Figure 13 | Characterization of the electrodeposits. a, TEM images of CeO_2 **b**, TEM images of $Ni(OH)_2$. Inset of **a** shows the SAED pattern and the size distribution of CeO_2 nanoparticles. Inset of **b** shows the SAED pattern of $Ni(OH)_2$. Scale bars: 10 nm.

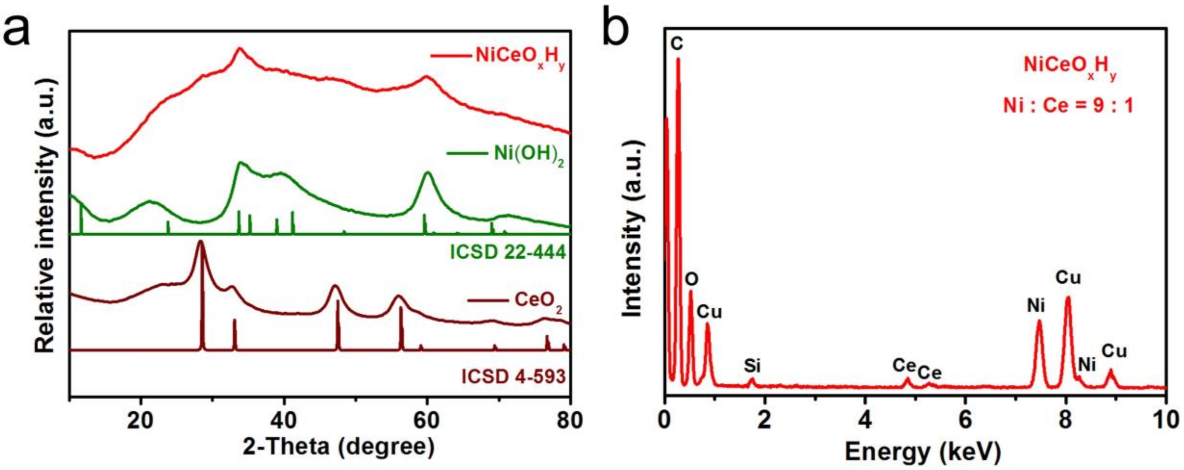

Supplementary Figure 14 | Characterization of the electrodeposits. a, Powder XRD patterns of CeO_2, $Ni(OH)_2$ and $NiCeO_xH_y$. **b**, TEM-EDS spectroscopy of $NiCeO_xH_y$.

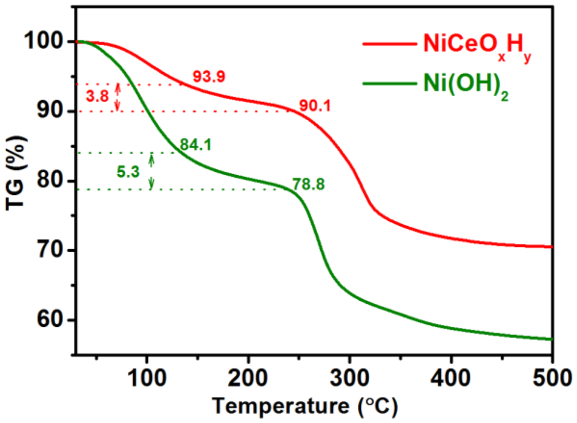

Supplementary Figure 15 | Thermogravimetric analysis of the NiCeO$_x$H$_y$ and Ni(OH)$_2$ powders.

The measurements were performed at a heating rate of 5 °C min^{-1} in air. The mass loss before 135 °C is caused by the evaporation of surface-adsorbed water, and the weight loss during 135–250 °C is attributed to the removal of lattice water and interlayer nitrate anions[4]. Weight loss at temperatures higher than 250 °C corresponds to phase transition from metal hydroxide to oxide[4,5]. Accordingly, the contents of Ni(OH)$_2$ and NiCeO$_x$H$_y$ in the samples are 78.8% and 90.1%, respectively.

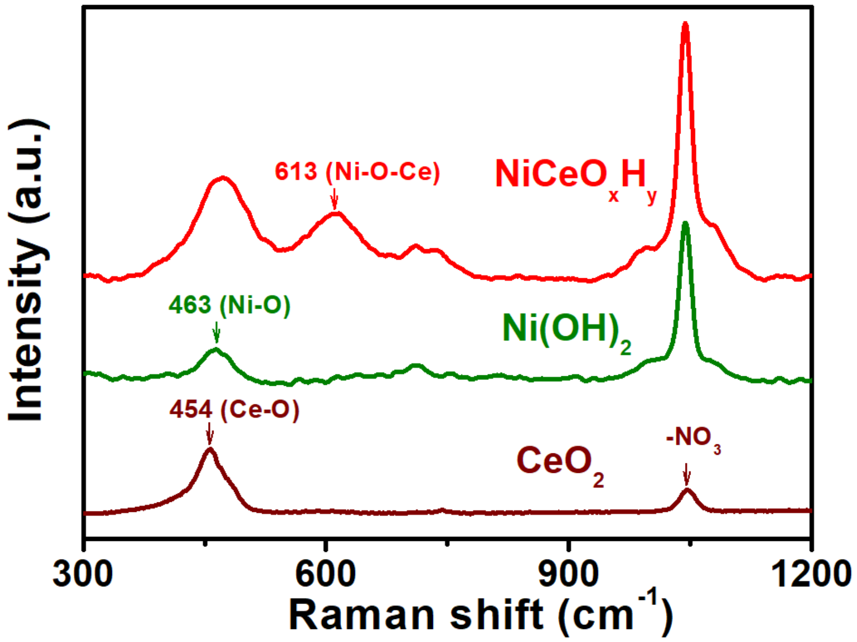

Supplementary Figure 16 | Characterization of the electrodeposits. Raman spectra of $Ni(OH)_2$, CeO_2 and $NiCeO_xH_y$ powders.

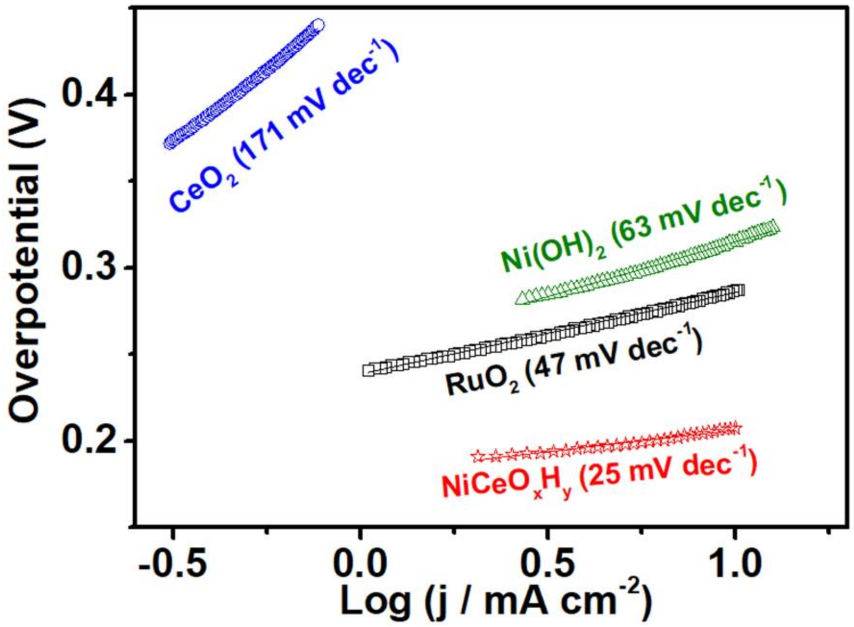

Supplementary Figure 17 | Tafel plots of different catalysts supported on rotating disk electrode (RDE) with a loading mass of 0.2 mg cm^{-2}. The datas derived from the OER polarization curves shown in Figure 5**a**.

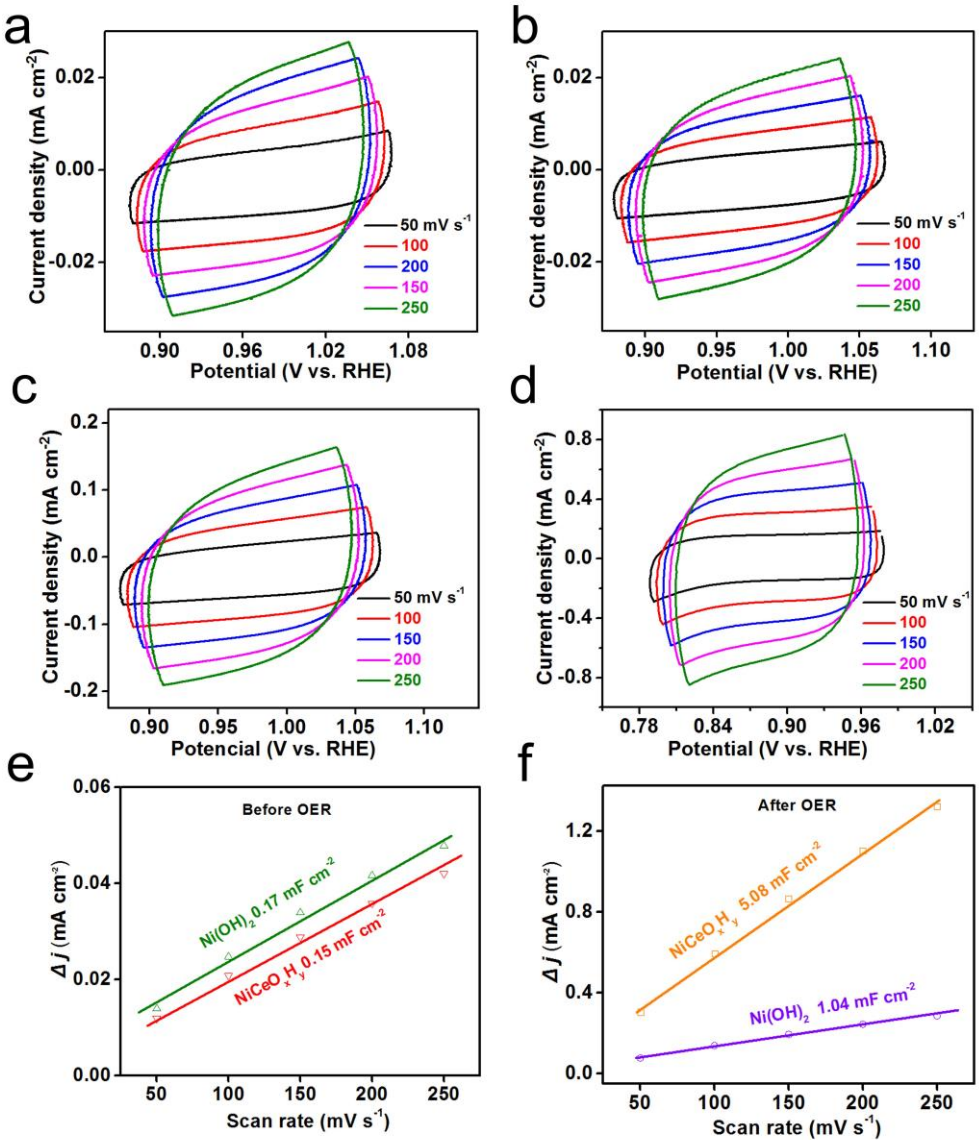

Supplementary Figure 18 | Comparison of electrochemically active surface area (ECSA) for electrodeposited $Ni(OH)_2$ and $NiCeO_xH_y$ catalyst before and after OER test. The double-layer capacitance measurements for determining ECSA were performed in 1 M KOH by RDE technique. Cyclic voltammograms of $Ni(OH)_2$ and $NiCeO_xH_y$. **a**,**b**, Before and **c**,**d**, after OER test. Charging current density difference plotted against scan rates **e**, before and **f**, after OER test. The linear slope is equivalent to twice of the double-layer capacitance C_{dl}. We use general specific capacitances of 0.04 mF cm^{-2} for all samples[6].

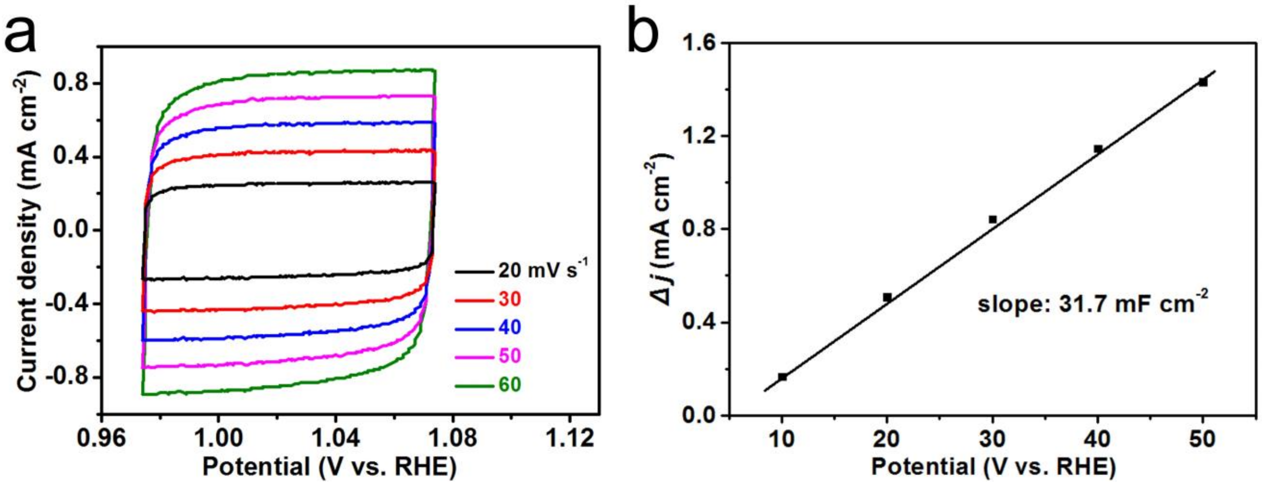

Supplementary Figure 19 | ECSA of commercial RuO_2 with a loading mass of 0.2 mg cm^{-2} in 1 M KOH tested by RDE technique within the non-Faradaic potential range. **a**, Cyclic voltammograms of RuO_2. **b**, Charging current density difference plotted against scan rates of RuO_2.

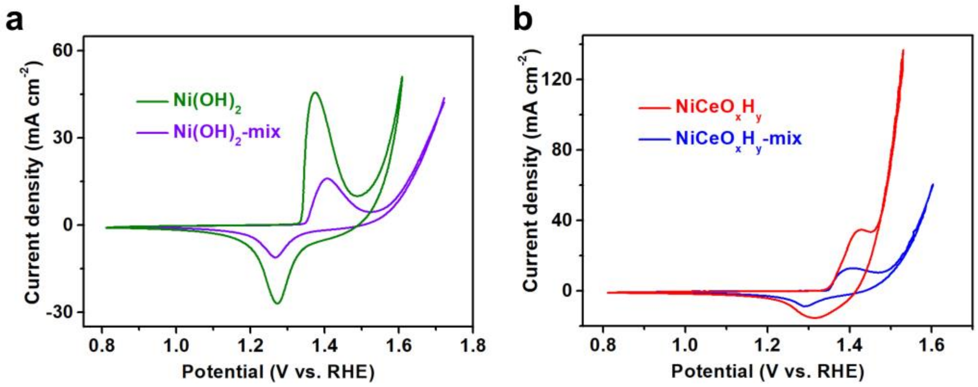

Supplementary Figure 20 | Electrocatalytic OER performance of electrodeposited and physically mixed electrodes. Voltametry (scan rate 10 mV s^{-1}) of **a**, $Ni(OH)_2$ and **b**, $NiCeO_xH_y$ coating on rotational glass carbon electrodes with mass loading of 0.2 mg cm^{-2} in 1.0 M KOH electrolyte. The mix electrodes were prepared by physically mixing the hydroxide electrocatalyst, carbon (Vulcan XC-72), and nafion (5 wt%) in a weight ratio of 50:40:10. For both $Ni(OH)_2$ and $NiCeO_xH_y$, the self-supporting electrodeposited electrodes outperform the powdery form mixed with carbon additive and polymeric binder.

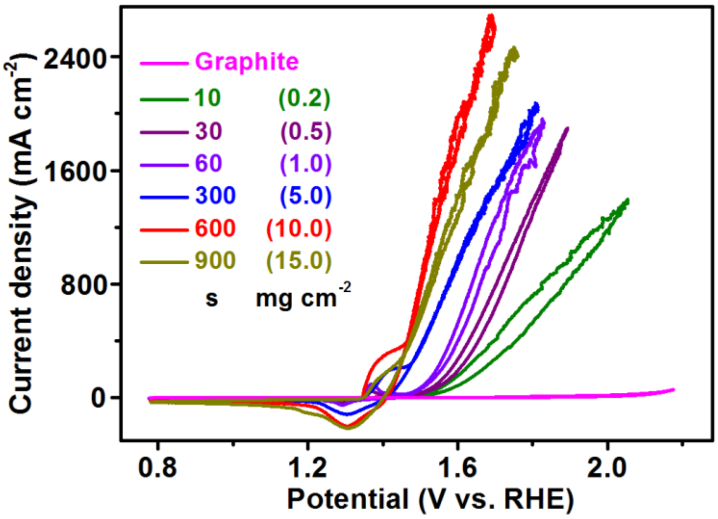

Supplementary Figure 21 | CV curves of $Ni_9Ce_1O_xH_y$/G electrodes with various loading mass in 1 M KOH. The $Ni_9Ce_1O_xH_y$/G electrodes were prepared by applying a current of 20 mA cm^{-2} in electrolyte containing 0.1 M total metal ion.

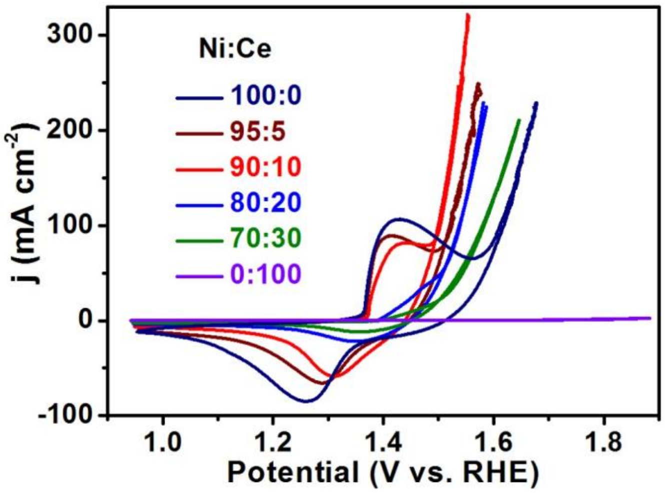

Supplementary Figure 22 | Effect of Ni:Ce ratio of NiCeO$_x$H$_y$/CP on electrochemical performance. Cyclic voltammetry curves in 1 M KOH solution. The NiCeO$_x$H$_y$/CP electrodes were prepared by applying 5 min deposition at a current of 20 mA cm^{-2}. The electrolyte contained 0.1 M total metal ion with different molar ratios of Ni:Ce.

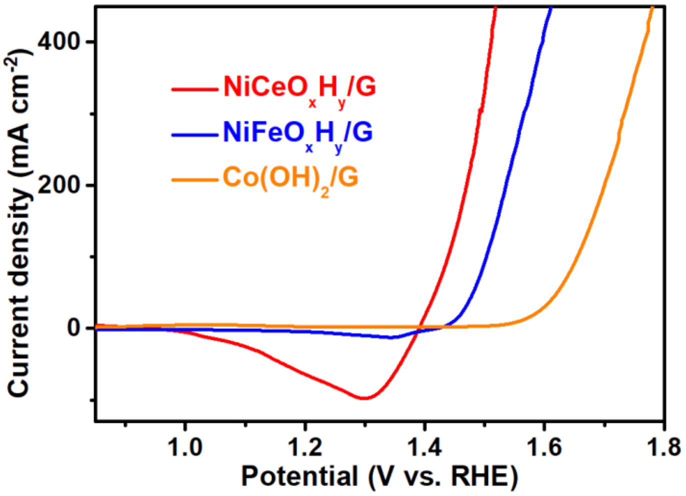

Supplementary Figure 23 | Electrocatalytic OER performance of NiCeO$_x$H$_y$, Co(OH)$_2$ and NiFeO$_x$H$_y$ electrodeposited on graphite. Polarization curves of the three electrodes in 1 M KOH solution at 10 mV s^{-1}. The atom ratio of Ni:Fe in the deposit solution is 1:1. All the electrodes were prepared by applying 5 min deposition at a current of 20 mA cm^{-2}. The overpotentials to reach a current density of 10 mA cm^{-2} are 177, 218 and 328 mV for NiCeO$_x$H$_y$, NiFeO$_x$H$_y$, and Co(OH)$_2$, respectively. Although inferior to NiCeO$_x$H$_y$, the Ni-Fe hybrid outperforms most of the reported NiFe-based hydroxide catalysts[7,8]. The Co-based electrode exhibits limited activity.

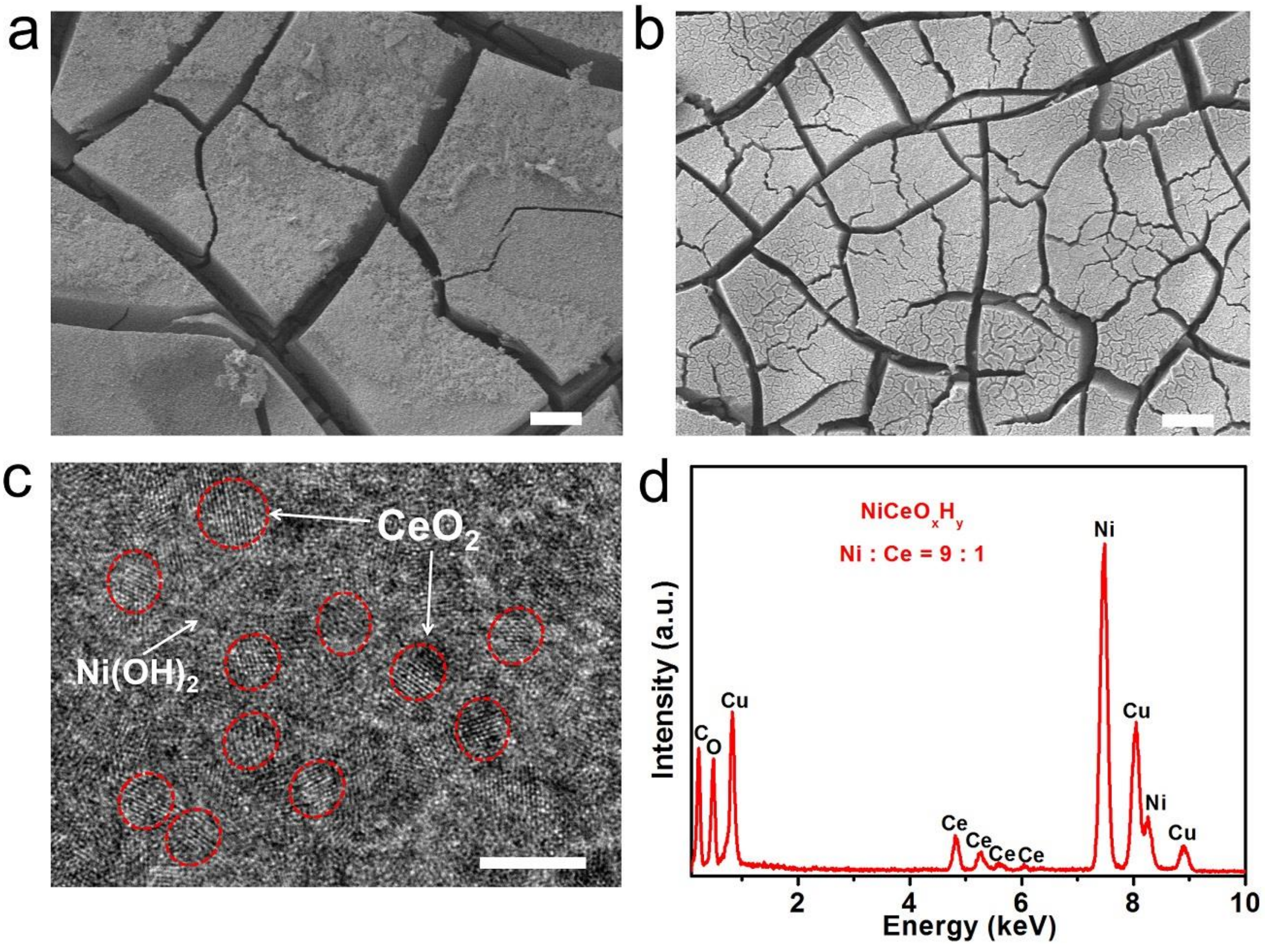

Supplementary Figure 24 | Morphologies and compositions of $NiCeO_xH_y$/graphite after the stability test (1000 mA cm^{-2}, 300 h). **a**, **b**, SEM images of the $NiCeO_xH_y$/graphite electrode **a**, at pristine state and **b**, after the OER test. **c**, TEM image and **d**, TEM-EDS spectrum of $NiCeO_xH_y$ after OER testing. Scale bars: **a**,**b**, 20 μm, **c**, 5 nm.

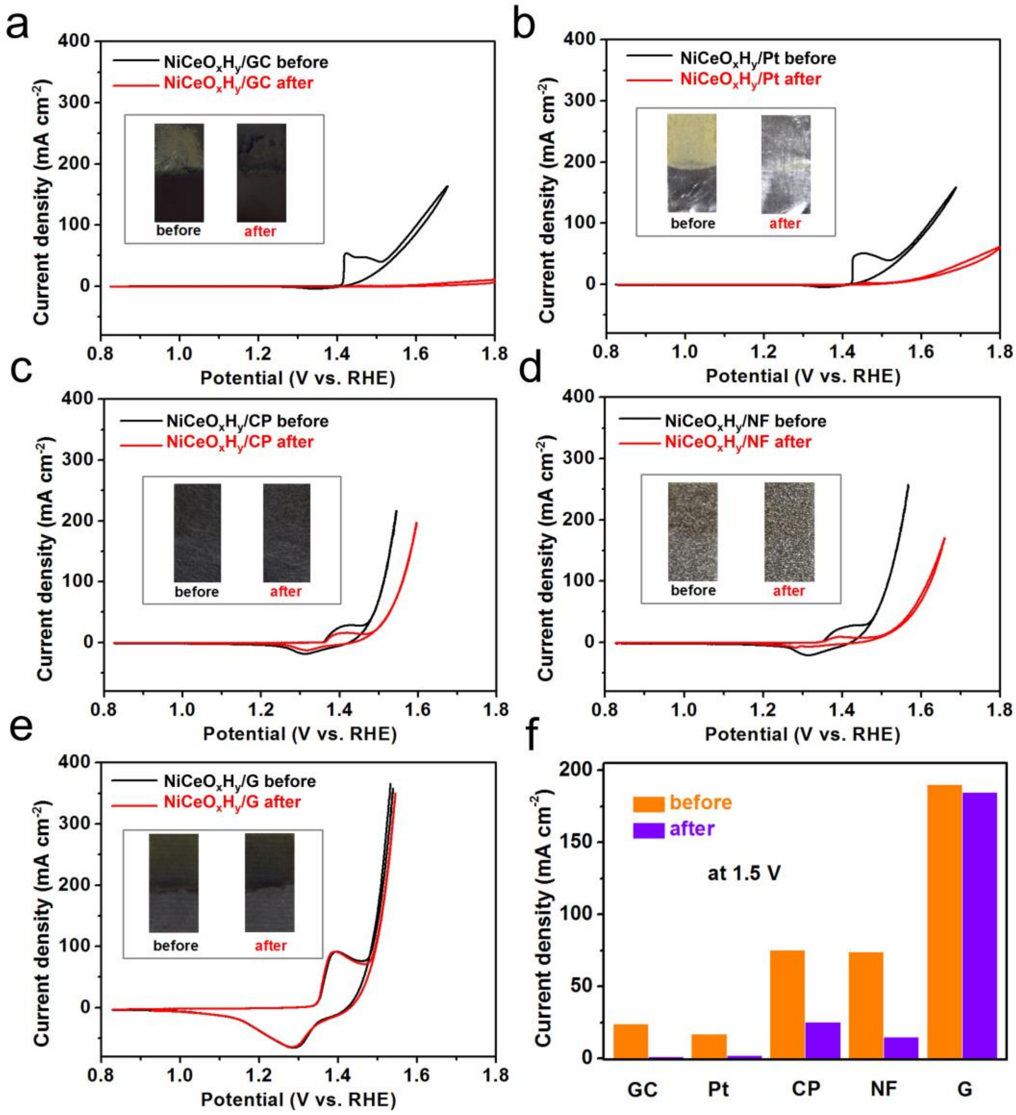

Supplementary Figure 25 | Adhesion strength of $NiCeO_xH_y$ on different substrates with a deposition of 20 mA cm^{-2} for 60s. Comparison the OER performance of $NiCeO_xH_y$ before and after ultrasonic treatment (80 W, 40 KHz) treatment for 300 s in water on substrate of **a**, GC, **b**, Pt, **c**, CP, **d**, NF, and **e**, G. The insets show the optical photographs of the electrodes before and after ultrasonic treatment. Obvious peeling off of deposits is observed on GC and Pt substrates. **f**, Comparison of the current densities collected at 1.5 V on different substrate-supported $NiCeO_xH_y$ electrodes. Loss of activity on GC, Pt, CP, and NF is likely ascribed to the falling off of the active mass during the ultrasonic treatment.

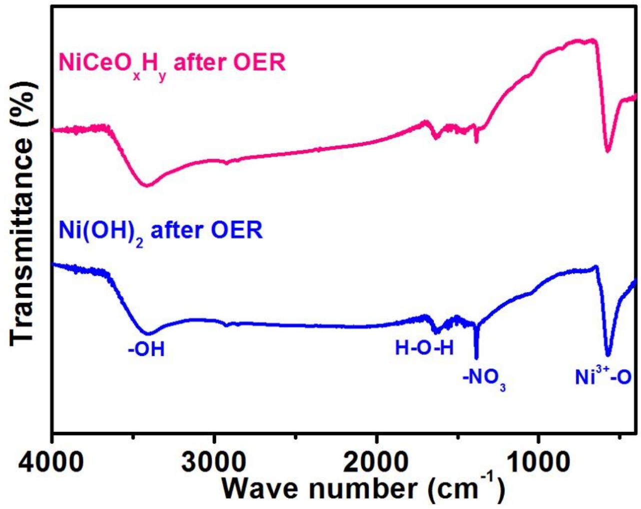

Supplementary Figure 26 | Compositions of electrodeposits after the stability test. FTIR of the electrodeposits after the stability test (1000 mA cm^{-2}, 300 h).

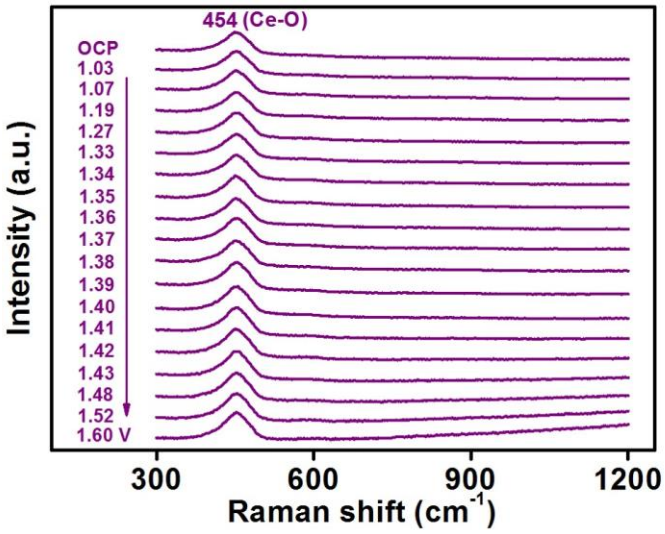

Supplementary Figure 27 | Structural evolution of CeO_2/G electrode during the OER. In situ Raman spectra collected from open circuit potential (OCP) to 1.6 V (vs. RHE) in 1 M KOH. There is no discernable change in Raman profile upon anodic scanning.

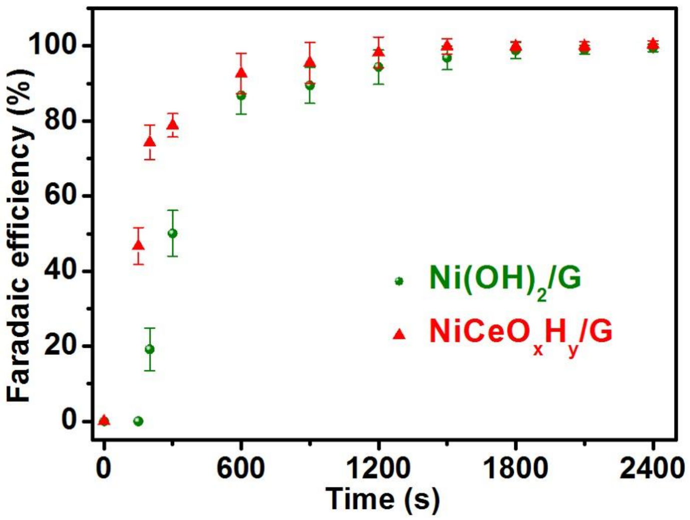

Supplementary Figure 28 | Comparison of Faraday efficiency of graphite-supported $Ni(OH)_2$ and $NiCeO_xH_y$. The mass loading is 10 mg cm^{-2}. Amount of evolved O_2 was measured using a drainage method in 1 M KOH solution. A constant current of 200 mA was applied to the working electrode. The low Faraday efficiency at early stage of the test suggests that there is a Ni(II)–Ni(III) transformation process. The higher OER Faraday efficiency of $NiCeO_xH_y$ than $Ni(OH)_2$ is indicative of promoted Ni(II)–Ni(III) transformation in $NiCeO_xH_y$. After the formation of stable catalytically active species, the Faraday efficiency approaches 100%.

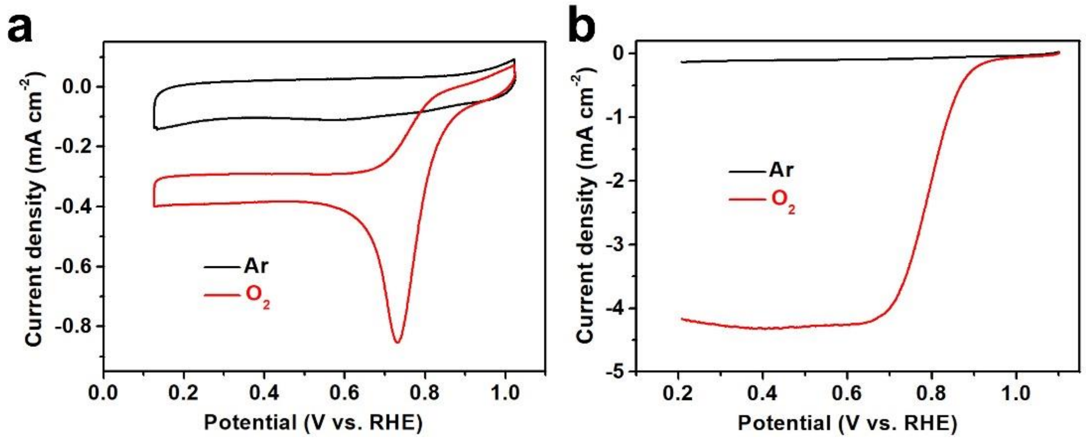

Supplementary Figure 29 | Electrocatalytic oxygen reduction performance of Mn_3O_4 in Ar- and O_2-saturated 0.1 M KOH solution. **a**, CV curves at a scan rate of 10 mV s^{-1}. **b**, Linear sweep voltammetry at 1600 rpm with a scan rate of 5 mV s^{-1}. The significantly enhanced cathodic currents in O_2-saturated electrolyte indicate ORR electrolysis. The onset and half potentials of the ORR are 0.95 and 0.8 V, respectively, among the highest values reported for Mn_3O_4 nanomaterials[9,10].

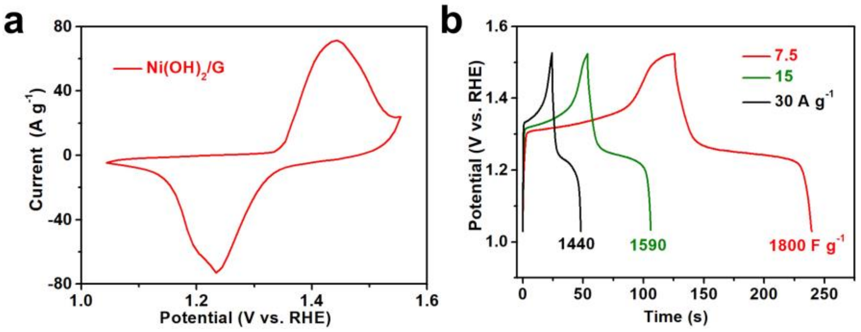

Supplementary Figure 30 | Supercapacitor performance of $Ni(OH)_2$/graphite electrode in 1 M KOH solution. **a**, CV curves at a scan rate of 10 mV s^{-1} of $Ni(OH)_2$/graphite electrode. **b**, Charge-discharge curves of $Ni(OH)_2$/graphite electrode at various current densities. The mass loading is 1 mg cm^{-2} and the specific gravimetric capacitance is based on the mass of $Ni(OH)_2$. The CV curve indicates a typical Faradaic redox process. The Coulombic efficiency estimated from CV currents is 99.1%, suggesting high reversibility of the electrodeposited $Ni(OH)_2$ on graphite. The $Ni(OH)_2$/graphite electrode delivers specific gravimetric capacitances of 1800, 1590, and 1440 F g^{-1} at currents of 7.5, 15, and 30 A g^{-1}, respectively.

Supplementary Table 1. Comparison of OER Activities for Different Catalysts[a]

Catalyst	Loading mass (mg cm^{-2})	η_{onset} (mV)	η @ 10 mA cm^{-2} (mV)	Activity @ η = 270 mV				Tafel slope (mV dec^{-1})	Ref.
				Geometric area-based (mA cm^{-2})	ECSA-based (mA cm^{-2})	Mass-based (A g^{-1})	TOF (s^{-1})		
$NiCeO_xH_y/G$	10.0	171	177	312	0.25	34.6	0.008	22	This work
$NiCeO_xH_y$	0.20	180	207	78	2.5	406.8	0.23	25	This work
$Ni(OH)_2$	0.20	270	310	1.2	0.2	7.1	0.004	63	This work
RuO_2	0.20	230	290	5.5	0.01	26	0.006	47	This work
FeCoW/Au Foam	0.21	182	191	-	~4	-	~0.31	37	11
NiFe/NF	-	-	215	72	6	-	0.075	28	12
NiFe LDH/GO	0.25	200	210	50	-	200	~0.15	39	13
NiCo-MOF/Cu Foam	0.2	-	189	18	-	36	~0.30	42	14
$NiCeO_x$–Au	-	210	290	8.0	-	-	~0.06	-	15
RuO_2	0.04	270	350	~2.0	~0.1	50	~0.06	94	16
IrO_x	0.21	260	340	~2.0	0.4	9.5	0.008	45	17
Ir/C-20 wt.%	0.25	220	270	25	-	100	-	40	7

[a]Unless stated, the catalysts were supported on glass carbon electrode. TOF is calculated on the basis of metal sites.

Supplementary References

1. Ren, H. M., Cai, C., Leng, C.B., Pang, S. F. & Zhang, Y. H. Nucleation kinetics in mixed $NaNO_3$/glycerol droplets investigated with the FTIR–ATR technique, *J. Phys. Chem. B* **120,** 2913-2920 (2016).
2. Kudin, K. N. *et al*. Raman spectra of graphite oxide and functionalized graphene sheets. *Nano Letters*, **8,** 36-41 (2008).
3. Wu, J. B., Lin, M. L., Cong, X., Liu, H. N. & Tan, P. H. Raman spectroscopy of graphene-based materials and its applications in related devices. *Chem. Soc. Rev.* **47,** 1822-1873 (2018).
4. Tian, X. Q. et al. Microwave-assisted non-aqueous homogenous precipitation of nanoball-like mesoporous α-$Ni(OH)_2$ as a precursor for NiO_x and its application as a pseudocapacitor. *J. Mater. Chem.* **22,** 8029-8035 (2012).
5. Wang, Y., Zhu, Q. & Zhang, H. G. Fabrication of β-$Ni(OH)_2$ and NiO hollow spheres by a facile template-free process. *Chem. Commun.* **41,** 5231-5233 (2005).
6. McCrory, C. C. L., Jung, S. H., Peters, J. C., & Jaramillo, T. F. Benchmarking heterogeneous electrocatalysts for the oxygen evolution reaction. *J. Am. Chem. Soc.* **135,** 16977-16987 (2013).
7. Gong, M. *et al.* An advanced Ni-Fe layered double hydroxide electrocatalyst for water oxidation. *J. Am. Chem. Soc.* **135,** 8452-8455 (2013).
8. Batchellor, A. S. & Boettcher, S. W. Pulse-electrodeposited Ni-Fe (oxy)hydroxide oxygen evolution electrocatalysts with high geometric and intrinsic activities at large mass loadings. *ACS Catal.* **5,** 6680-6689 (2015).

9. Li, C. S., Sun, Y., Lai, W. H., Wang, J. Z. & Chou S. L. Ultrafine Mn_3O_4 Nanowires/three-dimensional graphene/single-walled carbon nanotube composites: superior electrocatalysts for oxygen reduction and enhanced Mg/air batteries. *ACS. Appl. Mater. Interface* **8,** 27710-27719 (2016).
10. Duan, J. J. *et al*. Mesoporous hybrid material composed of Mn_3O_4 nanoparticles on nitrogen-doped graphene for highly efficient oxygen reduction reaction. *Chem. Commun.* **49,** 7705-7707 (2013).
11. Zhang, B. *et al.* Homogeneously dispersed multimetal oxygen-evolving catalysts. *Science* **352,** 333-337 (2016).
12. Lu, X. Y. & Zhao, C. Electrodeposition of hierarchically structured three-dimensional nickel-iron electrodes for efficient oxygen evolution at high current densities. *Nat. Commun.* **6,** 6616 (2015).
13. Long, X. *et al.* A strongly coupled graphene and FeNi double hydroxide hybrid as an excellent electrocatalyst for the oxygen evolution reaction. *Angew. Chem. Int. Ed.* **53,** 7584-7588 (2014).
14. Zhao, S. L. *et al.* Ultrathin metal-organic framework nanosheets for electrocatalytic oxygen evolution. *Nat. Energy* **1**, 16184 (2016).
15. Ng, D. J. W. *et al.* Gold-supported cerium-doped NiO_x catalysts for water oxidation. *Nat. Energy* **1**, 16053 (2016).
16. Xiao, Q. Q. *et al.* A high-performance electrocatalyst for oxygen evolution reactions based on electrochemical post-treatment of ultrathin carbon layer coated cobalt nanoparticles. *Chem. Commun.* **50,** 13019-13022 (2014).
17. Song F. & Hu, X. L. Exfoliation of layered double hydroxides for enhanced oxygen evolution catalysis. *Nat. Commun.* **5**, 4477 (2014).
