# Supplementary material for: Anion insertion enhanced electrodeposition of robust metal hydroxide/oxide electrodes for oxygen evolution
Source: Nat Commun. 2018 Jun 18;9:2373. doi: 10.1038/s41467-018-04788-3 (PMC6006371; doi:10.1038/s41467-018-04788-3)
Supplement: Supplementary file 2 — Description of Additional Supplementary Files [file 41467_2018_4788_MOESM2_ESM.pdf]

Description of Additional Supplementary Files

File Name: Supplementary Movie 1
Description: : Movie recording the water electrolysis process in the cell composed of a $NiCeO_xH_y$/G working electrode (W), a Pt foil counter electrode (C) and a Hg/HgO reference electrode (R).
